# Liver transcriptome data of *Esr1* knockout male rats reveals altered expression of genes involved in carbohydrate and lipid metabolism

**DOI:** 10.1016/j.dib.2018.12.089

**Published:** 2019-01-04

**Authors:** Vincentaben Khristi, Anamika Ratri, Subhra Ghosh, Shaon Borosha, Eddie Dai, V. Praveen Chakravarthi, M.A. Karim Rumi, Michael W. Wolfe

**Affiliations:** a Department of Pathology and Laboratory Medicine, University of Kansas Medical Center, Kansas City, KS 66160, United States; b Department of Molecular and Integrative Physiology, University of Kansas Medical Center, Kansas City, KS 66160, United States; c Institute for Reproduction and Perinatal Research, University of Kansas Medical Center, Kansas City, KS 66160, United States

## Abstract

Estrogens are traditionally considered to be female sex steroid hormones and most of the studies examining estrogen regulation of metabolic function in the liver have been conducted in females. However, the liver expresses high levels of estrogen receptor alpha (ESR1) in both males and females, which mediates the hepatic response to estrogens. In this data article, we investigated whether metabolic disorders in *Esr1* knockout (*Esr1-/-*) male rats were linked with loss of transcriptional regulation by ESR1 in liver. To identify the ESR1 regulated genes in the mutant liver, RNA-sequencing was performed on liver RNAs purified from young male rats. The raw data were analyzed using the CLC Genomics Workbench and high-quality RNA-sequencing reads were aligned to the *Rattus norvegicus* genome. Transcriptome data obtained from *Esr1-/-* liver RNAs were compared to that of wild type rats. Based on an absolute fold change of 2 with a *p*-value ≤ 0.05, a total of 618 differentially expressed genes were identified in the *Esr1-/-* male liver. Pathway analyses demonstrated that the majority of differentially expressed genes are regulators of carbohydrate and lipid metabolism in the liver. These differentially expressed genes and their potential roles were further examined in a companion manuscript, “Disruption of ESR1 alters the expression of genes regulating hepatic lipid and carbohydrate metabolism in male rats” (Khristi et al., 2018).

**Specifications table**

**Table:** 

Subject area	*Biology, Endocrinology*
More specific subject area	*Metabolic regulation in the liver*
Type of data	*RNA-seq data tables and figures*
How data were acquired	*RNA-Sequencing, Ingenuity Pathway Analysis*
Data format	*Normalized, filtered and analyzed data; Bioinformatic prediction*
Experimental factors	*Liver transcriptome profile in Esr1 knockout (Esr1-/-) male rats*
Experimental features	*Liver tissues were collected from 10-week-old wild type and Esr1-/- male rats. Total RNA was isolated, and cDNA-libraries were prepared for RNA-sequencing. RNA-seq raw data reads were analyzed using CLC Genomics Workbench. Differentially expressed genes were further analyzed for their involvement in carbohydrate and lipid metabolism by IPA.*
Data source location	*A basic science laboratory at the University of Kansas Medical Center, Kansas City, KS, USA.*
Data accessibility	*Raw data have not yet been submitted to any public repository.*
Related research article	*V. Khristi, A. Ratri, S. Ghosh, S. Borosha, E. Dai, R. Roy, et al., Disruption of ESR1 alters the expression of genes regulating hepatic lipid and carbohydrate metabolism in male rats, Endocrinology (2018), Under review*[1].

**Value of the data**

• This data article provides liver transcriptomic analyses of *Esr1-/-* male rats.
• Pathway analyses of the differentially expressed genes in the *Esr1*-/- liver show their involvement in carbohydrate and lipid metabolism.
• Differentially expressed genes are also linked to development of obesity, hepatic steatosis, and other liver diseases.

## 1 Data

**Fig. 1:**
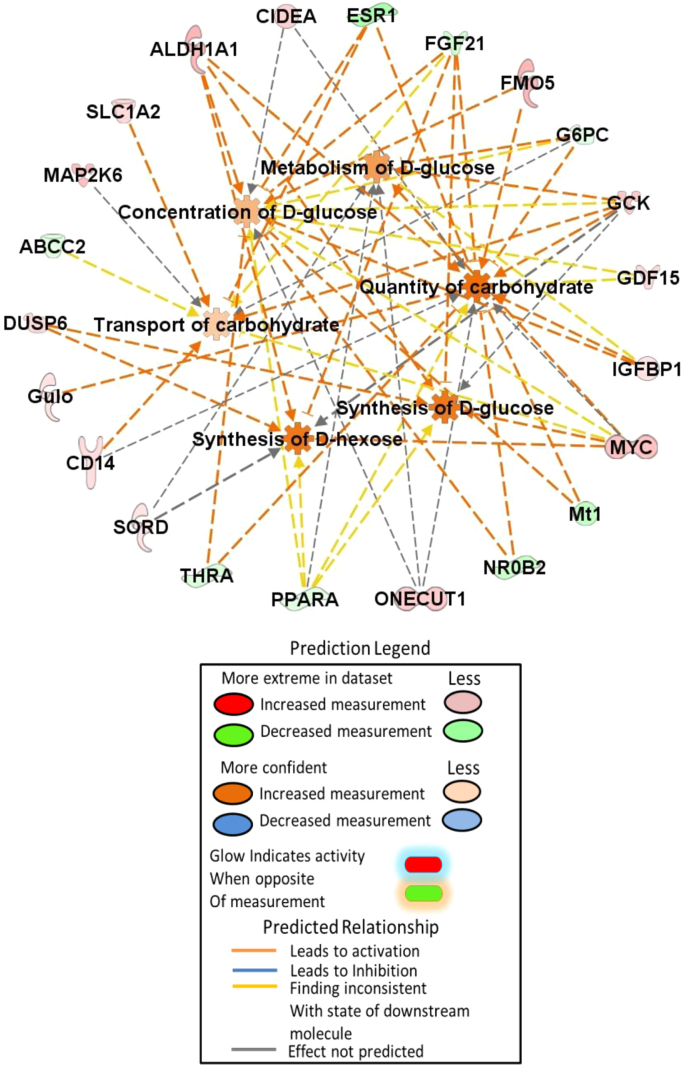
Mechanistic diagram of selected pathways involved in the carbohydrates metabolism.

**Fig. 2:**
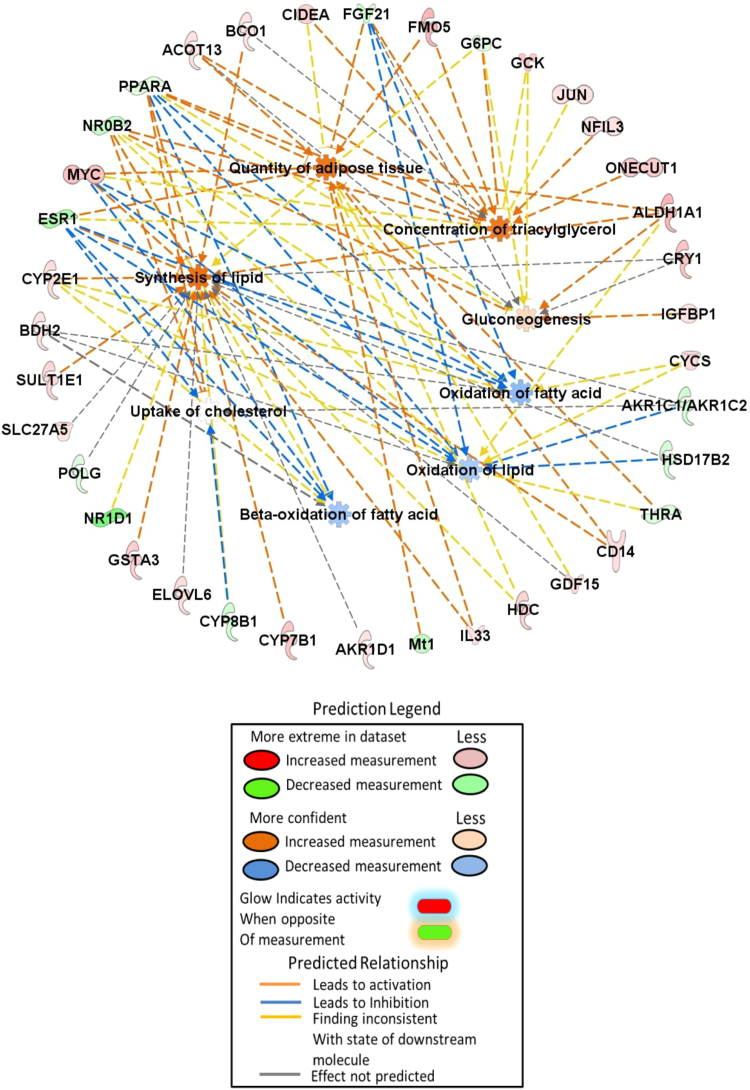
Mechanistic diagram of selected pathways involved in the synthesis and oxidation of lipids.

**Fig. 3:**
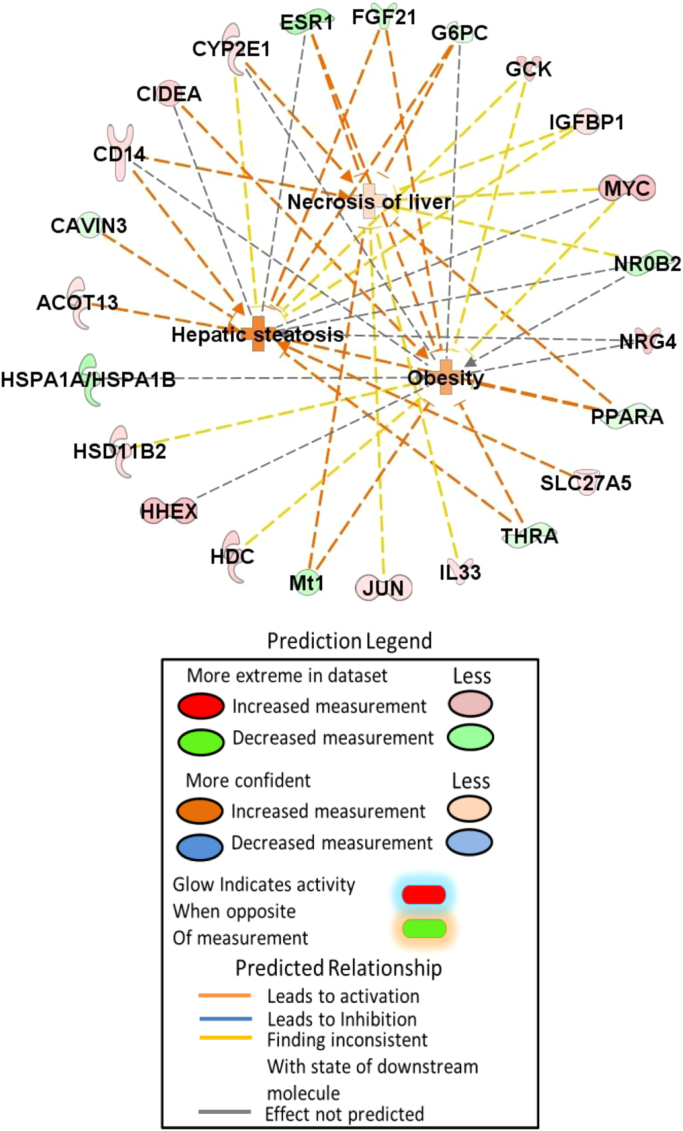
Mechanistic diagram of genes involved in hepatic steatosis, necrosis of the liver, and obesity.

In this data article, we present analyzed RNA-seq data showing the differentially expressed genes in the *Esr1-/-* male liver (Table S1). Bioinformatic analyses show that these differentially expressed genes are linked to pathways of carbohydrate metabolism (Table 1, Fig. 1), lipid metabolism (Table 2, Fig. 2) and hepatic diseases including hepatic steatosis, necrosis of the liver, and obesity (Fig. 3).

## 2 Experimental design, materials, and methods

### 2.1 *Esr1* knockout rats

The Holtzman Sprague-Dawley (HSD) *Esr1*-mutant rat model was generated by targeted deletion of exon 3 in the *Esr1* gene [2]. Deletion of exon 3 caused a frameshift and null mutation in the ESR1 coding sequence [2]. All animals were screened for the presence of the mutation by PCR using tail-tip DNA samples (REDExtract-N-Amp Tissue PCR Kit, Sigma-Aldrich) and primers targeting the flanking intron sequences [2]. All procedures were performed in accordance with the protocols approved by the University of Kansas Medical Center Animal Care and Use Committee.

### 2.2 Sample collection from wild type and *Esr1-/-* rats

Liver tissues were collected from 10 to 12-week-old *Esr1*-/- and age matched wild type male rats. The tissue samples were collected immediately after euthanization, cut into small species, snap frozen in liquid nitrogen, and stored at −80 °C until they were processed for RNA extraction. Total RNA from liver tissues was extracted using TRI Reagent (Millipore-Sigma) following the manufacturer׳s instructions. RNA quality was assessed using the Agilent Bioanalyzer and samples with a RIN score ≥ 9 were included in the RNA-seq library preparation.

### 2.3 Library preparation and RNA-sequencing

The library preparation and sequencing of RNA was performed at the Genome Sequencing facility of the University of Kansas Medical Center. Five hundred nanogram of liver total RNA was used for the RNA-seq library preparation. Libraries were prepared using a TruSeq Stranded mRNA kit (Illumina) following the manufacturer׳s instructions. Briefly, mRNA was enriched from total RNA by oligo-dT magnetic beads, purified, and chemically fragmented. The first strand of cDNA was synthesized using random hexamer primers and reverse transcriptase. Then, double stranded (ds) cDNA was generated by removing the RNA template and synthesizing a replacement strand, incorporating dUTP in place of dTTP. ds cDNA was purified from the second strand reaction mix by AMPure XP beads (Beckman Coulter). The cDNA ends were blunted and poly (A) tails were added to the 3’ ends. Finally, after ligation of indexing adaptors (Illumina), the suitable DNA fragments were selected for PCR amplification for 15 cycles. Three replicate cDNA libraries were prepared for each of the wild type and *Esr1-/-* groups and sequenced on an Illumina HiSeq. 2500 platform.

### 2.4 Analyses of the RNA-sequencing data

RNA-seq data were analyzed using CLC Genomics Workbench (Qiagen Bioinformatics). Raw reads of RNA-seq were analyzed as described in a previous publication [3]. Clean reads were obtained by removing low quality reads through trimming. High quality reads of liver RNA-seq were aligned to the *Rattus norvegicus* genome (Rn6, downloaded from NCBI database). RNA-seq data were mapped with the following parameters: (a) maximum number of allowed mismatches was set at 2; (b) minimum length and similarity fraction were set at 0.8; and (c) minimum number of hits per read was set at 10. Gene expression values were reported as RPKM (Reads Per Kilobase of transcript per Million mapped reads) [4]. In this study, gene expression values showing an absolute fold change of 2 with *p-*value ≤ 0.05 were considered differentially expressed. A total of 618 genes were differentially expressed in *Esr1-/-* liver, 410 downregulated and 208 upregulated (Table S1).

### 2.5 Pathway analysis of differentially expressed genes in *Esr1-/-* liver

Differentially expressed genes in the *Esr1-/-* liver were subjected to Ingenuity Pathway Analysis (IPA; Qiagen Bioinformatics). The pathways and genes involved in carbohydrate and lipid metabolism are listed in Tables 1 and 2. The selected pathways from carbohydrate metabolism shown elevated glucose level in *Esr1-/-* rats shown in Fig. 1. In lipid metabolism, synthesis of lipid and concentration of triglyceride were increased in *Esr1-/-* rats. In contrast, oxidation of lipid and fatty acid was lower in *Esr1-/-* rats (Fig. 2). The genes involved in hepatic steatosis and obesity are shown in Fig. 3.

**Table 1:** List of pathways involved in ‘Carbohydrate metabolism’.

**Diseases or functions annotation**	***p*****-value**	**Predicted activation state**	**Activation z-score**	**Molecules**	**# Molecules**
Gluconeogenesis	0.0000000		0.343	ACOT13, ALDH1A1, CRY1, FGF21, G6PC, GCK, IGFBP1, NR0B2, PPARA	9
Concentration of D-glucose	0.0000001		0.69	ALDH1A1, CIDEA, ESR1, FGF21, FMO5, G6PC, GCK, GDF15, IGFBP1, Mt1, MYC, NR0B2, ONECUT1, PPARA, THRA	15
Metabolism of D-glucose	0.0000001		1	G6PC, GCK, IGFBP1, MYC, ONECUT1, PPARA, SORD	7
Synthesis of D-hexose	0.0000023		1.735	ALDH1A1, DUSP6, FGF21, GCK, MYC, PPARA, SORD	7
Quantity of carbohydrate	0.0000024	Increased	2.257	ALDH1A1, CD14, CIDEA, ESR1, FGF21, FMO5, G6PC, GCK, GDF15, Gulo, IGFBP1, Mt1, MYC, NR0B2, ONECUT1, PPARA, THRA	17
Homeostasis of D-glucose	0.0000017			ACOT13, CIDEA, CRY1, FGF21, FMO5, G6PC, GCK, PPARA, SLC27A5, THRA	10
Transport of carbohydrate	0.000010		0.478	ABCC2, CD14, ESR1, FGF21, G6PC, GCK, MAP2K6, MYC, SLC1A2	9
Synthesis of D-glucose	0.0000023		1.735	ALDH1A1, DUSP6, FGF21, GCK, MYC, PPARA	6
Quantity of glucose-6-phosphate	0.000032			G6PC, GCK, MYC	3
Regulation of D-glucose	0.0000024			FGF21, MYC, PPARA	3
Utilization of D-glucose	0.000075			GCK, MYC, PPARA, THRA	4
Transport of monosaccharide	0.000119		0.555	ESR1, FGF21, G6PC, GCK, MAP2K6, MYC, SLC1A2	7
Phosphorylation of D-glucose	0.00021			G6PC, GCK	2
Metabolism of glucose-6-phosphate	0.0000057			G6PC, GCK	2
Transport of D-glucose	0.00044		0.555	ESR1, FGF21, GCK, MAP2K6, MYC, SLC1A2	6
Quantity of glycogen	0.0000100		1.172	FGF21, G6PC, GCK, MYC, PPARA	5
Synthesis of carbohydrate	0.000583		1.809	ALDH1A1, DUSP6, FGF2, G6PC, GCK, Gulo, IGFBP1, MYC, NR1D1, PPARA, SORD	11
Gluconeogenesis of hepatocytes	0.0000268			ALDH1A1, NR0B2	2
Import of D-glucose	0.0014		0	ESR1, FGF21, MYC, SLC1A2	4
Metabolism of carbohydrate	0.0000320		1.483	ALDH1A1, CYP2E1, DUSP6, FGF21, G6PC, GCK, Gulo, IGFBP1, MYC, NR1D1, ONECUT1, PPARA, SORD	13
Uptake of carbohydrate	0.00205		0.306	ABCC2, CD14, FGF21, G6PC, GCK, MYC, PPARA, SLC1A2	8
Synthesis of glycogen	0.0000560			G6PC, GCK, IGFBP1, NR1D1	4
Quantity of lactic acid	0.00214			GCK, MYC, PPARA	3
Production of lactic acid	0.0000750			GCK, MYC, PPARA	3

**Table 2:** List of pathways involved in ‘Lipid metabolism’.

**Diseases or functions annotation**	***p*****-value**	**Predicted activation state**	**Activation z-score**	**Molecules**	**# Molecules**
Synthesis of terpenoid	0.0000000		1.169	AKR1C1/AKR1C2, AKR1D1, ALDH1A1, BCO1, CRY1, CYP7B1, CYP8B1, ESR1, G6PC, GDF15, GSTA3, HDC, HSD17B2, NR1D1, POLG, PPARA, SLC27A5, SULT1E1	18
Metabolism of terpenoid	0.0000000		0.342	ADH7, AKR1C1/AKR1C2, AKR1D1, ALDH1A1, BCO1, CYP2E1, CYP7B1, CYP8B1, ESR1, G6PC, GSTA3, HDC, HSD11B2, HSD17B2, NR0B2, SLC27A5, SULT1E1, UGT2B11	18
Quantity of steroid	0.0000000		0.547	ABCC2, ACOT13, BCO1, CRY1, CYP8B1, ESR1, FGF21, FMO5, G6PC, GCK, Gulo, HSD11B2, IL33, JUN, NFIL3, NR0B2, POLG, PPARA, SLC1A2, SULT1E1, THRA, ZBTB16	22
Concentration of lipid	0.0000000		1.111	ABCC2, ACOT13, ALDH1A1, BCO1, CD14, CIDEA, CRY1, CYP2E1, CYP8B1, EFNA5, ESR1, FGF21, FMO5, G6PC, GCK, Gulo, HSD11B2, IL33, JUN, MYC, NFIL3, NR0B2, ONECUT1, POLG, PPARA, SLC1A2, SULT1E1, THRA, ZBTB16	29
Synthesis of steroid	0.0000000		0.601	AKR1C1/AKR1C2, AKR1D1, CRY1, CYP7B1, CYP8B1, ESR1, G6PC, GDF15, GSTA3, HDC, HSD17B2, NR1D1, POLG, PPARA, SLC27A5, SULT1E1	16
Steroid metabolism	0.0000000		−0.594	AKR1C1/AKR1C2, AKR1D1, CYP2E1, CYP7B1, CYP8B1, ESR1, G6PC, GSTA3, HDC, HSD11B2, HSD17B2, NR0B2, SLC27A5, SULT1E1, UGT2B11	15
Concentration of cholesterol	0.0000000		1.352	ACOT13, BCO1, CYP8B1, ESR1, FGF21, FMO5, G6PC, GCK, Gulo, IL33, JUN, NFIL3, NR0B2, PPARA, SLC1A2, THRA	16
Synthesis of lipid	0.0000000	Increased	2.092	AKR1C1/AKR1C2, AKR1D1, ALDH1A1, BCO1, CD14, CRY1, CYP2E1, CYP7B1, CYP8B1, ELOVL6, ESR1, G6PC, GDF15, GSTA3, HDC, HSD17B2, IL33, MYC, NR0B2, NR1D1, POLG, PPARA, SLC27A5, SULT1E1	24
Homeostasis of lipid	0.0000000			AKR1C1/AKR1C2, CIDEA, CYP7B1, CYP8B1, FGF21, G6PC, GCK, Mt1, NR0B2, NR1D1, NR1D2, PPARA	12
Oxidation of lipid	0.0000000		−0.47	AKR1C1/AKR1C2, ALDH1A1, BDH2, CYCS, CYP2E1, ESR1, FGF21, HSD17B2, MYC, NR0B2, PPARA, THRA	12
Synthesis of bile acid	0.0000000		−0.132	AKR1D1, CYP7B1, CYP8B1, NR1D1, PPARA, SLC27A5	6
Concentration of acyl glycerol	0.00000		1.388	ACOT13, BCO1, CIDEA, ESR1, FGF21, FMO5, G6PC, GCK, JUN, MYC, NFIL3, NR0B2, ONECUT1, PPARA, THRA	15
Concentration of triacylglycerol	0.00000		1.651	ACOT13, BCO1, CIDEA, ESR1, FGF21, FMO5, G6PC, GCK, JUN, MYC, NFIL3, NR0B2, ONECUT1, PPARA	14
Uptake of cholesterol	0.0000158		0	AKR1C1/AKR1C2, CYP8B1, ESR1, NR0B2, PPARA	5
Concentration of bile acid	0.000019			ABCC2, CYP8B1, ESR1, NR0B2	4
Quantity of ketone body	0.0000334			ACOT13, FGF21, GCK, PPARA	4
Inactivation of glucocorticoid	0.0000352			AKR1D1, HSD11B2	2
Inactivation of lipid	0.0000566			AKR1D1, HSD11B2, SULT1E1	3
Secretion of lipid	0.0000772		0.927	ABCC2, CIDEA, ESR1, HSD17B2, MAP2K6, NFIL3, PPARA, SULT1E1	8
Absorption of cholesterol	0.0000842			AKR1C1/AKR1C2, CYP8B1, NR0B2, PPARA	4
Uptake of lipid	0.000206		−0.7	AKR1C1/AKR1C2, CD14, CYP8B1, ESR1, NR0B2, PPARA, SLC27A5	7
Production of ketone body	0.000348			FGF21, GCK	2
Metabolism of retinoid	0.000453			ADH7, ALDH1A1, BCO1, CYP2E1	4
Concentration of fatty acid	0.000463		1.773	ACOT13, CIDEA, CYP2E1, EFNA5, FGF21, G6PC, GCK, MYC, PPARA	9
Synthesis of ketone body	0.00052			PPARA, SLC27A5	2
Metabolism of triacylglycerol	0.000537			ALDH1A1, CYP2E1, G6PC, NR0B2, PPARA	5
Abnormal quantity of lipid	0.00055			ACOT13, CYP8B1, ESR1, FGF21, GCK, NR0B2	6
Hydroxylation of lipid	0.000607			CYP2E1, CYP4A22, CYP7B1	3
Conversion of lipid	0.000622		0.905	AKR1C1/AKR1C2, CYP2E1, HSD11B2, HSD17B2, Mt1, PPARA	6
Metabolism of sterol	0.000856			AKR1D1, CYP7B1, CYP8B1, HSD17B2, NR0B2	5
Regulation of lipid	0.00138			FGF21, HSD11B2, PPARA	3
Modification of long-chain acyl-coenzyme A	0.00154			ELOVL6, PPARA	2
Activation of lipid	0.00161			FGF21, HSD11B2, SLC27A5	3
Synthesis of sterol	0.00183		1	CYP7B1, CYP8B1, HDC, PPARA	4
Hydroxylation of fatty acid	0.00187			CYP2E1, CYP4A22	2
Transport of lipid	0.00209		0.386	ABCC2, AKR1C1/AKR1C2, CD14, GDF15, IL33, PPARA, SLC27A5	7
Fatty acid metabolism	0.00277		0.767	ABCC2, AKR1C1/AKR1C2, CD14, CYP2E1, CYP4A22, DBP, ELOVL6, GDF15, IL33, MYC, PPARA, SLC27A5	12
Abnormal quantity of bile salt	0.00306			CYP8B1, NR0B2	2
Uptake of bile salt	0.00306			NR0B2, PPARA	2
Metabolism of bile acid	0.00352			AKR1C1/AKR1C2, SLC27A5	2
Regulation of steroid	0.00352			HSD11B2, PPARA	2
Homeostasis of bile salt	0.00452			CYP8B1, NR0B2	2
Homeostasis of cholesterol	0.005			AKR1C1/AKR1C2, CYP7B1, G6PC, NR1D1	4
Conjugation of lipid	0.00506			SLC27A5, UGT2B11	2
Transport of steroid	0.00529		−0.106	ABCC2, AKR1C1/AKR1C2, GDF15, IL33, PPARA	5
Metabolism of cholesterol	0.00531			AKR1D1, CYP7B1, CYP8B1, NR0B2	4

## 3 Statistical analysis

RNA-sequencing included three cDNA libraries in each group. Each library was prepared from pooled total RNA of two individual rats. Differentially expressed genes were identified by using CLC Genomics Workbench as described in a previous publication [5].
