# Supplementary material for: Liver transcriptome data of Esr1 knockout male rats reveals altered expression of genes involved in carbohydrate and lipid metabolism
Source: Data Brief. 2019 Jan 4;22:771–80. doi: 10.1016/j.dib.2018.12.089 (PMC6330359; doi:10.1016/j.dib.2018.12.089)
Supplement: Supplementary file 2 — Supplementary material [file mmc2.docx]

**Table 1. List of differentially expressed genes in *Esr1* knockout male liver**

| **Gene symbol** | **Chrom.** | **Region** | **Max group mean** | **Log2 fold change** | **Fold change** | ***p*-value** | **FDR**  ***p*-value** | **Bonferroni** |
| --- | --- | --- | --- | --- | --- | --- | --- | --- |
| A2m | 4 | 154309426..154359137 | 0.89 | 1.28 | 2.43 | 0.004 | 0.400 | 1.000 |
| A2ml1 | 4 | complement(161866078..161907767) | 0.44 | -1.56 | -2.95 | 0.001 | 0.130 | 1.000 |
| AABR07000398.1 | 1 | 11963836..11968939 | 10881.45 | -7.63 | -198.14 | 0.000 | 0.000 | 0.000 |
| AABR07000658.1 | 1 | complement(21145360..21146846) | 0.45 | -1.45 | -2.72 | 0.008 | 0.550 | 1.000 |
| AABR07001477.1 | 1 | complement(46740305..46744647) | 0.01 | -5.03 | -32.57 | 0.040 | 1.000 | 1.000 |
| AABR07001591.1 | 1 | 53841653..53844518 | 0.17 | -1.30 | -2.47 | 0.040 | 0.940 | 1.000 |
| AABR07001634.1 | 1 | complement(54439977..54441579) | 1.01 | 2.62 | 6.15 | 0.000 | 0.001 | 0.030 |
| AABR07001807.1 | 1 | complement(58339947..58345450) | 0.07 | -1.48 | -2.79 | 0.020 | 0.850 | 1.000 |
| AABR07004397.2 | 1 | complement(141209402..141210059) | 2.12 | -3.48 | -11.17 | 0.000 | 0.000 | 0.008 |
| AABR07006097.1 | 1 | complement(219752584..219758885) | 0.10 | -1.72 | -3.30 | 0.020 | 0.780 | 1.000 |
| AABR07006672.1 | 1 | complement(245816791..245817809) | 0.14 | -1.75 | -3.37 | 0.050 | 1.000 | 1.000 |
| AABR07007055.1 | 1 | complement(278476630..278480271) | 0.46 | -1.64 | -3.11 | 0.001 | 0.220 | 1.000 |
| AABR07013425.1 | 2 | complement(236822072..236823145) | 0.12 | -2.08 | -4.22 | 0.030 | 0.860 | 1.000 |
| AABR07013746.1 | 2 | 254227523..254250415 | 1.49 | -1.09 | -2.14 | 0.010 | 0.700 | 1.000 |
| AABR07014259.1 | 14 | 6221085..6223394 | 0.71 | 2.95 | 7.72 | 0.040 | 0.970 | 1.000 |
| AABR07015055.1 | 14 | 46523589..46523999 | 3429.32 | -7.68 | -204.43 | 0.000 | 0.000 | 0.000 |
| AABR07015066.1 | 14 | 46588406..46588816 | 3426.01 | -7.70 | -208.29 | 0.000 | 0.000 | 0.000 |
| AABR07015078.1 | 14 | 46649971..46650381 | 3432.03 | -7.66 | -202.30 | 0.000 | 0.000 | 0.000 |
| AABR07015078.2 | 14 | complement(46649976..46650359) | 2520.56 | -8.91 | -480.47 | 0.000 | 0.000 | 0.000 |
| AABR07015080.2 | 14 | 46653035..46653445 | 3427.15 | -7.67 | -203.24 | 0.000 | 0.000 | 0.000 |
| AABR07016578.3 | 14 | 105083805..105085260 | 0.23 | 2.16 | 4.48 | 0.030 | 0.890 | 1.000 |
| AABR07017145.1 | 15 | 12756403..12796711 | 0.10 | 3.44 | 10.82 | 0.020 | 0.760 | 1.000 |
| AABR07018132.1 | 15 | complement(42270040..42271443) | 0.06 | -3.05 | -8.26 | 0.040 | 0.930 | 1.000 |
| AABR07018269.2 | 15 | complement(49561460..49619581) | 2.38 | 1.10 | 2.15 | 0.010 | 0.660 | 1.000 |
| AABR07019085.1 | 15 | complement(87404841..87450319) | 0.47 | -1.16 | -2.23 | 0.020 | 0.770 | 1.000 |
| AABR07021492.1 | 13 | complement(74611854..74617038) | 1.15 | -2.14 | -4.41 | 0.000 | 0.050 | 1.000 |
| AABR07021544.1 | 13 | complement(77818965..77821312) | 1.94 | 1.29 | 2.45 | 0.040 | 1.000 | 1.000 |
| AABR07025316.1 | 16 | 33373423..33376382 | 0.11 | -2.22 | -4.67 | 0.005 | 0.450 | 1.000 |
| AABR07026912.2 | 17 | 4567620..4568359 | 0.31 | -2.16 | -4.47 | 0.040 | 0.940 | 1.000 |
| AABR07027272.1 | 17 | complement(22252020..22263289) | 0.39 | -1.63 | -3.10 | 0.002 | 0.280 | 1.000 |
| AABR07027902.1 | 17 | 48436720..48459292 | 0.08 | -3.05 | -8.28 | 0.010 | 0.640 | 1.000 |
| AABR07028995.2 | 10 | complement(1701449..1702704) | 0.48 | -3.33 | -10.08 | 0.005 | 0.440 | 1.000 |
| AABR07031399.1 | 18 | complement(12224514..12284188) | 0.15 | -1.25 | -2.38 | 0.040 | 0.990 | 1.000 |
| AABR07031762.1 | 18 | complement(33142195..33156047) | 1.80 | 3.47 | 11.04 | 0.003 | 0.330 | 1.000 |
| AABR07032821.1 | 18 | complement(84443531..84444218) | 0.92 | 2.25 | 4.77 | 0.020 | 0.800 | 1.000 |
| AABR07033465.1 | 11 | 22852240..22852431 | 1.54 | 6.22 | 74.44 | 0.030 | 0.880 | 1.000 |
| AABR07033540.1 | 11 | complement(26720424..26721540) | 0.14 | -2.35 | -5.08 | 0.010 | 0.620 | 1.000 |
| AABR07034592.1 | 11 | 80304977..80310498 | 0.02 | -2.79 | -6.93 | 0.020 | 0.770 | 1.000 |
| AABR07035780.3 | 12 | 22356942..22361493 | 0.06 | -2.05 | -4.13 | 0.050 | 1.000 | 1.000 |
| AABR07035780.5 | 12 | 22340574..22351622 | 0.06 | -1.98 | -3.93 | 0.030 | 0.910 | 1.000 |
| AABR07036007.1 | 12 | 30441055..30444565 | 1.07 | 2.91 | 7.52 | 0.010 | 0.700 | 1.000 |
| AABR07041109.1 | X | complement(119532513..119532965) | 2.11 | 1.30 | 2.46 | 0.030 | 0.850 | 1.000 |
| AABR07042859.1 | 19 | complement(12402521..12403635) | 0.20 | -2.43 | -5.39 | 0.007 | 0.540 | 1.000 |
| AABR07043190.1 | 19 | complement(26352497..26354487) | 0.80 | -2.23 | -4.70 | 0.001 | 0.130 | 1.000 |
| AABR07043365.1 | 19 | 27832781..27835780 | 0.27 | -2.96 | -7.77 | 0.000 | 0.040 | 1.000 |
| AABR07043510.1 | 19 | complement(28986730..28990676) | 0.99 | -2.36 | -5.12 | 0.000 | 0.040 | 1.000 |
| AABR07043822.1 | 19 | 41654261..41655649 | 3.53 | 1.19 | 2.29 | 0.007 | 0.510 | 1.000 |
| AABR07043825.1 | 19 | complement(42455947..42516312) | 0.41 | -2.53 | -5.79 | 0.020 | 0.800 | 1.000 |
| AABR07044001.4 | 19 | complement(52051267..52061809) | 0.44 | -1.64 | -3.11 | 0.010 | 0.690 | 1.000 |
| AABR07045047.1 | 20 | complement(32797689..32798870) | 0.34 | -1.27 | -2.40 | 0.040 | 1.000 | 1.000 |
| AABR07048637.1 | 5 | complement(85852471..85857989) | 0.06 | -1.86 | -3.62 | 0.010 | 0.610 | 1.000 |
| AABR07050487.1 | 5 | 167061794..167087844 | 0.02 | -3.62 | -12.30 | 0.007 | 0.520 | 1.000 |
| AABR07051310.1 | 3 | complement(4762148..4766540) | 0.04 | -5.39 | -42.02 | 0.030 | 0.890 | 1.000 |
| AABR07051562.1 | 3 | complement(17080904..17081510) | 0.65 | 2.96 | 7.76 | 0.010 | 0.600 | 1.000 |
| AABR07052458.1 | 3 | complement(55012951..55025095) | 7.94 | 1.02 | 2.03 | 0.004 | 0.390 | 1.000 |
| AABR07056503.1 | 7 | 27438351..27442171 | 0.02 | -4.46 | -22.08 | 0.004 | 0.430 | 1.000 |
| AABR07058192.1 | 7 | 104192167..104197688 | 0.10 | -1.64 | -3.12 | 0.010 | 0.690 | 1.000 |
| AABR07059004.1 | 4 | 848551..870720 | 0.10 | -2.84 | -7.15 | 0.005 | 0.480 | 1.000 |
| AABR07059632.3 | 4 | complement(28220398..28224858) | 0.66 | -1.04 | -2.05 | 0.002 | 0.230 | 1.000 |
| AABR07059675.1 | 4 | 31704747..31705121 | 0.07 | -3.69 | -12.89 | 0.040 | 0.930 | 1.000 |
| AABR07060133.1 | 4 | complement(58408730..58485447) | 0.11 | -3.61 | -12.21 | 0.001 | 0.220 | 1.000 |
| AABR07060678.1 | 4 | 89247300..89252822 | 0.03 | -2.63 | -6.18 | 0.010 | 0.640 | 1.000 |
| AABR07063424.1 | 6 | 30638733..30639143 | 3436.07 | -7.69 | -206.34 | 0.000 | 0.000 | 0.000 |
| AABR07063893.1 | 6 | 54438436..54443719 | 0.08 | -2.21 | -4.62 | 0.010 | 0.640 | 1.000 |
| AABR07064590.1 | 6 | complement(86160266..86161333) | 0.56 | -1.13 | -2.19 | 0.050 | 1.000 | 1.000 |
| AABR07066188.1 | 9 | 4621425..4624425 | 3.04 | 1.91 | 3.75 | 0.002 | 0.240 | 1.000 |
| AABR07066529.1 | 9 | 10787455..10789519 | 0.96 | -1.10 | -2.15 | 0.020 | 0.750 | 1.000 |
| AABR07068043.1 | 9 | complement(79293740..79309691) | 1.36 | -2.01 | -4.02 | 0.000 | 0.020 | 1.000 |
| AABR07068044.2 | 9 | complement(79323408..79325098) | 1.24 | -1.94 | -3.85 | 0.001 | 0.220 | 1.000 |
| AABR07068127.1 | 9 | 84550603..84552964 | 0.24 | -1.36 | -2.57 | 0.030 | 0.890 | 1.000 |
| AABR07071598.1 | 8 | complement(123828751..123829749) | 0.09 | -3.96 | -15.56 | 0.008 | 0.550 | 1.000 |
| AABR07072207.1 | 10 | 105564535..105565162 | 0.15 | -4.70 | -25.91 | 0.040 | 0.990 | 1.000 |
| AABR07073181.1 | 5 | 155812105..155885829 | 0.32 | -1.25 | -2.38 | 0.004 | 0.410 | 1.000 |
| Abcb9 | 12 | 37923528..37958363 | 0.36 | 1.18 | 2.27 | 0.030 | 0.910 | 1.000 |
| Abcc2 | 1 | 263554453..263613252 | 43.11 | -1.09 | -2.14 | 0.001 | 0.160 | 1.000 |
| Abcc8 | 1 | complement(102110708..102191287) | 1.01 | -1.16 | -2.23 | 0.000 | 0.080 | 1.000 |
| Abhd10 | 11 | 60054408..60066739 | 0.65 | -1.11 | -2.16 | 0.030 | 0.860 | 1.000 |
| Abhd2 | 1 | 140998240..141087405 | 32.28 | -1.34 | -2.53 | 0.000 | 0.002 | 0.140 |
| Abo3 | 3 | 5118956..5126901 | 0.31 | 3.07 | 8.41 | 0.006 | 0.490 | 1.000 |
| AC098750.2 | 2 | complement(188596245..188597639) | 1.04 | -2.04 | -4.10 | 0.001 | 0.200 | 1.000 |
| AC106681.1 | 19 | complement(10261345..10267141) | 0.37 | 2.64 | 6.24 | 0.030 | 0.860 | 1.000 |
| AC112350.1 | 3 | complement(114182444..114183114) | 0.17 | -3.20 | -9.19 | 0.005 | 0.460 | 1.000 |
| AC115420.1 | 4 | 157416904..157418184 | 0.78 | -2.07 | -4.20 | 0.040 | 0.950 | 1.000 |
| AC124896.1 | 7 | complement(36495480..36499784) | 13.48 | 1.20 | 2.30 | 0.003 | 0.370 | 1.000 |
| AC127640.1 | 1 | complement(75455599..75462453) | 0.04 | -2.42 | -5.35 | 0.010 | 0.600 | 1.000 |
| AC128353.1 | 13 | 37507604..37513125 | 0.30 | 1.65 | 3.14 | 0.007 | 0.510 | 1.000 |
| AC128960.1 | 17 | complement(78835332..78835594) | 0.39 | -2.51 | -5.69 | 0.020 | 0.740 | 1.000 |
| AC135645.2 | 3 | 80965783..80968447 | 0.30 | 2.36 | 5.13 | 0.040 | 1.000 | 1.000 |
| Ackr2 | 8 | 130538651..130542227 | 0.26 | -2.70 | -6.48 | 0.001 | 0.160 | 1.000 |
| Acot13 | 17 | 42241159..42253649 | 13.99 | 1.02 | 2.03 | 0.001 | 0.190 | 1.000 |
| Acpp | 8 | complement(112834707..112884077) | 1.38 | -1.65 | -3.13 | 0.000 | 0.040 | 1.000 |
| Acsm5 | 1 | 189233141..189272172 | 4.81 | 1.70 | 3.25 | 0.000 | 0.000 | 0.010 |
| Adam11 | 10 | 90731148..90749217 | 3.60 | -1.06 | -2.09 | 0.003 | 0.350 | 1.000 |
| Adamts9 | 4 | complement(124660262..124858250) | 0.38 | -1.22 | -2.32 | 0.001 | 0.130 | 1.000 |
| Adgrg2 | X | complement(36930186..37003642) | 0.04 | -1.88 | -3.67 | 0.030 | 0.910 | 1.000 |
| Adh1 | 2 | 243550627..243687857 | 428.66 | 1.60 | 3.04 | 0.001 | 0.180 | 1.000 |
| Adh7 | 2 | 243502073..243516532 | 13.51 | 2.42 | 5.36 | 0.000 | 0.000 | 0.000 |
| Afmid | 10 | 106828981..106844314 | 91.56 | 1.14 | 2.20 | 0.000 | 0.040 | 1.000 |
| Ago2 | 7 | complement(114339434..114380613) | 0.53 | -1.05 | -2.07 | 0.030 | 0.890 | 1.000 |
| Akap4 | X | complement(16296735..16306078) | 0.06 | -3.16 | -8.95 | 0.006 | 0.490 | 1.000 |
| Akr1c2 | 17 | complement(69388335..69404341) | 29.87 | -1.75 | -3.37 | 0.000 | 0.070 | 1.000 |
| Akr1d1 | 4 | 65110746..65143941 | 365.58 | 1.04 | 2.06 | 0.040 | 0.930 | 1.000 |
| Aldh1a1 | 1 | 238222521..238264330 | 42.98 | 2.57 | 5.96 | 0.000 | 0.000 | 0.000 |
| Aldh1a7 | 1 | complement(240562423..240601744) | 3.09 | 1.70 | 3.25 | 0.010 | 0.690 | 1.000 |
| Aldh1b1 | 5 | 61382351..61387358 | 8.73 | 1.03 | 2.05 | 0.006 | 0.500 | 1.000 |
| Aldoc | 10 | 65586504..65590126 | 0.15 | 2.38 | 5.21 | 0.020 | 0.790 | 1.000 |
| Amdhd1 | 7 | complement(34361633..34406318) | 19.72 | -1.87 | -3.65 | 0.000 | 0.000 | 0.000 |
| Amigo1 | 2 | 210977938..210982579 | 0.80 | -1.07 | -2.10 | 0.002 | 0.270 | 1.000 |
| Anln | 8 | complement(23337621..23390773) | 0.04 | -5.93 | -61.09 | 0.008 | 0.560 | 1.000 |
| Apc2 | 7 | complement(12258468..12275609) | 0.05 | -1.28 | -2.42 | 0.030 | 0.870 | 1.000 |
| Arfgef3 | 1 | complement(13838707..13915594) | 0.07 | -1.53 | -2.89 | 0.020 | 0.780 | 1.000 |
| Armt1 | 1 | 40982862..41006371 | 4.53 | 1.05 | 2.08 | 0.000 | 0.040 | 1.000 |
| Atp8b4 | 3 | complement(118591880..118959850) | 0.13 | -1.11 | -2.16 | 0.040 | 0.980 | 1.000 |
| Atxn1 | 17 | 19249952..19533814 | 0.64 | -1.26 | -2.40 | 0.006 | 0.490 | 1.000 |
| Bche | 2 | complement(171100140..171196395) | 1.06 | 1.07 | 2.10 | 0.001 | 0.220 | 1.000 |
| Bcl6 | 11 | 80255790..80279075 | 8.31 | -2.00 | -4.00 | 0.000 | 0.000 | 0.001 |
| Bco1 | 19 | 49637016..49673808 | 2.51 | 1.06 | 2.09 | 0.020 | 0.730 | 1.000 |
| Bdh2 | 2 | 240461505..240482145 | 7.26 | 1.00 | 2.01 | 0.003 | 0.310 | 1.000 |
| Begain | 6 | complement(132936970..132972511) | 0.04 | -2.07 | -4.20 | 0.040 | 1.000 | 1.000 |
| Bhlha15 | 12 | complement(12403385..12407561) | 0.86 | -2.06 | -4.16 | 0.000 | 0.001 | 0.080 |
| Birc6 | 6 | complement(21950100..22092346) | 1.61 | -1.04 | -2.06 | 0.006 | 0.480 | 1.000 |
| C2cd4d | 2 | 195603599..195604624 | 0.74 | 1.08 | 2.12 | 0.040 | 0.980 | 1.000 |
| Cabp5 | 1 | 75693232..75706069 | 0.07 | -5.32 | -39.86 | 0.020 | 0.790 | 1.000 |
| Cacnb3 | 7 | 140315368..140324902 | 0.25 | -1.62 | -3.08 | 0.004 | 0.370 | 1.000 |
| Casr | 11 | 67188630..67258771 | 0.03 | -2.41 | -5.32 | 0.020 | 0.740 | 1.000 |
| Cbx4 | 10 | complement(108190995..108196217) | 2.89 | -1.10 | -2.14 | 0.006 | 0.490 | 1.000 |
| Ccdc126 | 4 | 78981987..79003053 | 4.73 | 1.05 | 2.07 | 0.001 | 0.180 | 1.000 |
| Cd14 | 18 | complement(29560365..29562153) | 35.47 | 1.24 | 2.37 | 0.000 | 0.005 | 0.330 |
| Cd209a | 12 | complement(2341960..2347543) | 0.42 | 1.58 | 2.98 | 0.020 | 0.790 | 1.000 |
| Cd36 | 4 | 14001761..14249749 | 3.94 | 1.95 | 3.86 | 0.000 | 0.000 | 0.002 |
| Cdc14a | 2 | complement(219302577..219458271) | 0.55 | 1.13 | 2.19 | 0.020 | 0.770 | 1.000 |
| Cdc20 | 5 | complement(137260739..137265015) | 0.75 | -1.38 | -2.60 | 0.005 | 0.440 | 1.000 |
| Cdh24 | 15 | complement(33278713..33285779) | 0.25 | -1.10 | -2.14 | 0.050 | 1.000 | 1.000 |
| Cenpf | 13 | complement(108132499..108178609) | 0.08 | -1.39 | -2.62 | 0.010 | 0.710 | 1.000 |
| Chad | 10 | 82292110..82296651 | 0.22 | -1.95 | -3.87 | 0.006 | 0.490 | 1.000 |
| Chn1 | 3 | complement(60513077..60611924) | 0.11 | -1.49 | -2.81 | 0.030 | 0.910 | 1.000 |
| Chst15 | 1 | complement(204245645..204322544) | 0.30 | -1.15 | -2.21 | 0.010 | 0.700 | 1.000 |
| Chst9 | 18 | complement(6834365..7081356) | 1.29 | 1.00 | 2.00 | 0.020 | 0.840 | 1.000 |
| Ciart | 2 | complement(197987705..197991574) | 0.43 | -2.74 | -6.69 | 0.004 | 0.370 | 1.000 |
| Cib2 | 8 | complement(59123079..59139946) | 1.31 | -3.18 | -9.04 | 0.000 | 0.080 | 1.000 |
| Cidea | 18 | 63098144..63108051 | 5.08 | 1.67 | 3.19 | 0.001 | 0.110 | 1.000 |
| Cish | 8 | 116054465..116060723 | 9.95 | 4.37 | 20.73 | 0.000 | 0.000 | 0.000 |
| Cit | 12 | complement(46334665..46494174) | 0.10 | -1.08 | -2.11 | 0.050 | 1.000 | 1.000 |
| Clca2 | 2 | complement(250954397..250981623) | 0.08 | -4.07 | -16.85 | 0.000 | 0.070 | 1.000 |
| Cmah | 17 | complement(42583945..42640221) | 0.10 | -1.84 | -3.59 | 0.030 | 0.880 | 1.000 |
| Col12a1 | 8 | complement(87040799..87158368) | 0.29 | -1.15 | -2.22 | 0.005 | 0.440 | 1.000 |
| Col25a1 | 2 | 235596123..235715057 | 0.30 | 1.56 | 2.96 | 0.030 | 0.880 | 1.000 |
| Col26a1 | 12 | 22835019..22980603 | 0.13 | -2.33 | -5.04 | 0.010 | 0.690 | 1.000 |
| Col6a6 | 8 | complement(114353825..114449956) | 0.11 | -1.27 | -2.41 | 0.030 | 0.910 | 1.000 |
| Cpa3 | 2 | complement(105016626..105047984) | 0.24 | 1.84 | 3.59 | 0.020 | 0.830 | 1.000 |
| Crtc1 | 16 | 20740826..20794039 | 0.97 | -1.04 | -2.06 | 0.020 | 0.850 | 1.000 |
| Cry1 | 7 | 24534615..24634097 | 4.82 | 2.02 | 4.06 | 0.000 | 0.000 | 0.000 |
| Cux2 | 12 | 40018937..40219291 | 0.15 | -1.45 | -2.74 | 0.020 | 0.840 | 1.000 |
| Cxcl14 | 17 | 9109731..9117750 | 1.85 | 1.08 | 2.11 | 0.020 | 0.780 | 1.000 |
| Cycs | 18 | complement(62476383..62476700) | 17.07 | 1.30 | 2.45 | 0.010 | 0.690 | 1.000 |
| Cyp2c24 | 1 | 258074860..258139176 | 0.45 | -1.15 | -2.22 | 0.040 | 0.980 | 1.000 |
| Cyp2e1 | 1 | 213511874..213535542 | 1090.87 | 1.23 | 2.35 | 0.010 | 0.700 | 1.000 |
| Cyp4a8 | 5 | complement(133978954..134008255) | 2.42 | -2.34 | -5.08 | 0.000 | 0.000 | 0.000 |
| Cyp7b1 | 2 | complement(102701903..102871257) | 6.50 | 1.93 | 3.80 | 0.000 | 0.000 | 0.003 |
| Cyp8b1 | 8 | complement(130548418..130550388) | 94.89 | -1.76 | -3.40 | 0.000 | 0.006 | 0.500 |
| Dact1 | 6 | 93740586..93749809 | 0.59 | 2.80 | 6.97 | 0.000 | 0.005 | 0.320 |
| Dact2 | 1 | complement(56159426..56168351) | 0.61 | -1.22 | -2.32 | 0.004 | 0.420 | 1.000 |
| Dbp | 1 | 101687855..101692846 | 12.50 | -2.01 | -4.04 | 0.000 | 0.040 | 1.000 |
| Dcbld2 | 11 | complement(44241111..44292884) | 0.25 | -1.69 | -3.23 | 0.002 | 0.280 | 1.000 |
| Ddhd1 | 15 | complement(19965187..20032191) | 7.63 | 1.40 | 2.65 | 0.000 | 0.001 | 0.040 |
| Ddx11 | 9 | 114111580..114132492 | 0.30 | -1.02 | -2.02 | 0.040 | 0.970 | 1.000 |
| Dll1 | 1 | complement(57318708..57327379) | 0.95 | -1.21 | -2.32 | 0.008 | 0.560 | 1.000 |
| Dnah1 | 16 | complement(7345978..7408265) | 0.05 | -1.20 | -2.31 | 0.020 | 0.760 | 1.000 |
| Dnajb7 | 7 | complement(122642740..122644054) | 0.20 | -1.63 | -3.10 | 0.040 | 0.930 | 1.000 |
| Dock3 | 8 | complement(115629583..115981910) | 0.49 | -1.01 | -2.02 | 0.010 | 0.640 | 1.000 |
| Dppa4 | 11 | complement(55234545..55240241) | 0.40 | 2.43 | 5.37 | 0.020 | 0.770 | 1.000 |
| Dusp6 | 7 | 41475163..41479392 | 10.15 | 1.43 | 2.70 | 0.000 | 0.000 | 0.020 |
| Dyrk1b | 1 | 85112247..85120839 | 1.46 | -1.03 | -2.05 | 0.001 | 0.220 | 1.000 |
| Edil3 | 2 | 17616401..18128512 | 0.11 | 1.99 | 3.98 | 0.010 | 0.630 | 1.000 |
| Efemp1 | 14 | 113202419..113295014 | 0.50 | -1.33 | -2.51 | 0.009 | 0.590 | 1.000 |
| Efna5 | 9 | complement(110057155..110225486) | 3.50 | -1.06 | -2.08 | 0.006 | 0.480 | 1.000 |
| Egfl6 | X | 29430831..29488739 | 0.10 | -2.22 | -4.65 | 0.020 | 0.730 | 1.000 |
| Eml1 | 6 | 132383337..132450393 | 0.30 | -1.08 | -2.12 | 0.030 | 0.890 | 1.000 |
| Entpd7_2 | 1 | 263448633..263490308 | 0.58 | -1.15 | -2.21 | 0.007 | 0.550 | 1.000 |
| Ep300 | 7 | 122818975..122861296 | 1.10 | -1.02 | -2.03 | 0.007 | 0.540 | 1.000 |
| Epb41l4a | 18 | complement(26570647..26658892) | 0.41 | -1.19 | -2.27 | 0.020 | 0.770 | 1.000 |
| Epb42 | 3 | complement(112965653..112985318) | 0.31 | 1.72 | 3.30 | 0.001 | 0.210 | 1.000 |
| Epcam | 6 | complement(11281964..11298216) | 45.26 | -1.51 | -2.85 | 0.010 | 0.700 | 1.000 |
| Epha2 | 5 | 159845774..159874206 | 3.26 | -1.29 | -2.45 | 0.002 | 0.270 | 1.000 |
| Erich5 | 7 | 73222730..73242899 | 0.72 | 1.55 | 2.94 | 0.040 | 0.930 | 1.000 |
| Esm1 | 2 | 45104305..45113131 | 0.42 | -1.71 | -3.27 | 0.030 | 0.850 | 1.000 |
| Esr1 | 1 | 41192824..41594796 | 2.24 | -3.22 | -9.30 | 0.000 | 0.000 | 0.000 |
| Etv3 | 2 | 186872520..186886592 | 3.42 | -1.13 | -2.19 | 0.000 | 0.010 | 1.000 |
| Exoc7 | 10 | complement(105163754..105182834) | 0.68 | -4.74 | -26.73 | 0.000 | 0.010 | 0.770 |
| F2rl2 | 2 | 26240385..26245533 | 0.07 | -2.18 | -4.52 | 0.030 | 0.880 | 1.000 |
| F3 | 2 | 225310624..225322272 | 0.35 | -1.07 | -2.09 | 0.030 | 0.880 | 1.000 |
| Faim_1 | 8 | 106816152..106827580 | 0.37 | -6.61 | -97.70 | 0.005 | 0.440 | 1.000 |
| Fam13a | 4 | complement(89183768..89281222) | 3.95 | 1.19 | 2.28 | 0.000 | 0.080 | 1.000 |
| Fam151a | 5 | 126334803..126347770 | 0.04 | -3.05 | -8.26 | 0.030 | 0.910 | 1.000 |
| Fam212b | 2 | 208170652..208185965 | 0.04 | -1.90 | -3.74 | 0.040 | 1.000 | 1.000 |
| Fat4 | 2 | 125752130..125879387 | 0.05 | -1.39 | -2.63 | 0.010 | 0.690 | 1.000 |
| Fcgbpl1 | 1 | 85058548..85096346 | 0.03 | -4.04 | -16.49 | 0.010 | 0.620 | 1.000 |
| Fez1 | 8 | 39305128..39350270 | 0.34 | 1.64 | 3.12 | 0.010 | 0.710 | 1.000 |
| Fgf1 | 18 | 32273770..32359824 | 1.92 | 1.61 | 3.05 | 0.000 | 0.000 | 0.020 |
| Fgf21 | 1 | complement(101595579..101596822) | 2.81 | -2.02 | -4.07 | 0.007 | 0.540 | 1.000 |
| Filip1 | 8 | complement(87256264..87419564) | 0.14 | -1.33 | -2.51 | 0.005 | 0.440 | 1.000 |
| Flrt1 | 1 | complement(222344713..222350173) | 0.10 | -1.55 | -2.92 | 0.020 | 0.830 | 1.000 |
| Fmo5 | 2 | 199796881..199823927 | 64.19 | 2.51 | 5.71 | 0.000 | 0.000 | 0.000 |
| Fmod | 13 | 50873605..50885563 | 0.20 | -1.68 | -3.20 | 0.002 | 0.300 | 1.000 |
| Fndc1 | 1 | 47605262..47687504 | 0.04 | -1.75 | -3.37 | 0.040 | 0.950 | 1.000 |
| Fndc3b | 2 | complement(113112902..113345577) | 4.01 | -1.05 | -2.07 | 0.000 | 0.100 | 1.000 |
| Foxk1 | 12 | complement(14180029..14244316) | 0.89 | -1.10 | -2.14 | 0.004 | 0.400 | 1.000 |
| Foxm1 | 4 | 161685258..161696305 | 0.35 | -1.10 | -2.14 | 0.010 | 0.610 | 1.000 |
| Foxq1 | 17 | complement(34224818..34226453) | 0.09 | 2.95 | 7.72 | 0.040 | 0.970 | 1.000 |
| Fras1 | 14 | complement(14439082..14689554) | 0.54 | 2.75 | 6.73 | 0.000 | 0.000 | 0.002 |
| Frmd4b | 4 | complement(129658840..129846642) | 4.11 | -1.05 | -2.07 | 0.000 | 0.070 | 1.000 |
| Frs3 | 9 | complement(15297895..15306465) | 0.84 | -1.06 | -2.09 | 0.020 | 0.790 | 1.000 |
| Fuom | 1 | 141832774..141836250 | 1.19 | 1.26 | 2.39 | 0.020 | 0.770 | 1.000 |
| G6pc | 10 | 89285855..89296213 | 177.48 | -1.19 | -2.28 | 0.009 | 0.590 | 1.000 |
| Gadd45a | 4 | complement(97782512..97784842) | 12.48 | 1.38 | 2.60 | 0.000 | 0.030 | 1.000 |
| Gadd45g | 17 | complement(13391467..13393243) | 24.74 | 1.15 | 2.22 | 0.006 | 0.510 | 1.000 |
| Gck | 14 | complement(86148928..86190659) | 14.92 | 2.00 | 4.00 | 0.000 | 0.000 | 0.000 |
| Gda | 1 | complement(238982392..239057732) | 11.30 | 1.45 | 2.73 | 0.000 | 0.003 | 0.160 |
| Gdf15 | 16 | 20555395..20557978 | 3.02 | 1.37 | 2.58 | 0.030 | 0.910 | 1.000 |
| Gfra1 | 1 | complement(279207586..279277339) | 2.06 | -1.16 | -2.23 | 0.001 | 0.210 | 1.000 |
| Gfra3 | 18 | complement(27491845..27520295) | 1.86 | 1.20 | 2.29 | 0.001 | 0.200 | 1.000 |
| Gimap4 | 4 | 78320190..78327144 | 0.51 | 1.51 | 2.85 | 0.010 | 0.600 | 1.000 |
| Gimap7 | 4 | 78354335..78358503 | 0.48 | 1.17 | 2.25 | 0.050 | 1.000 | 1.000 |
| Gins1 | 3 | 146695344..146716816 | 0.60 | 1.40 | 2.63 | 0.020 | 0.850 | 1.000 |
| Glipr1l2 | 7 | complement(54795260..54823956) | 0.29 | 1.54 | 2.91 | 0.020 | 0.830 | 1.000 |
| Glt1d1 | 12 | complement(32666635..32740732) | 2.99 | 1.27 | 2.41 | 0.005 | 0.440 | 1.000 |
| Gna14 | 1 | 235166718..235347937 | 1.17 | 1.14 | 2.21 | 0.006 | 0.480 | 1.000 |
| Gnai1 | 4 | 13405136..13485883 | 0.73 | -1.65 | -3.13 | 0.001 | 0.140 | 1.000 |
| Gpat2 | 3 | 119994537..120003090 | 0.18 | -1.80 | -3.49 | 0.010 | 0.680 | 1.000 |
| Gpr158 | 17 | 88215834..88651476 | 0.08 | 2.24 | 4.73 | 0.020 | 0.770 | 1.000 |
| Gpr21 | 3 | 21764377..21767300 | 0.01 | -5.68 | -51.37 | 0.020 | 0.830 | 1.000 |
| Gpr22 | 6 | complement(51011426..51019407) | 0.01 | -2.88 | -7.38 | 0.020 | 0.800 | 1.000 |
| Greb1l | 18 | 1723565..1784233 | 0.30 | -1.15 | -2.22 | 0.020 | 0.720 | 1.000 |
| Gria3 | X | 127562660..127829753 | 1.18 | -1.96 | -3.90 | 0.000 | 0.000 | 0.001 |
| Grik5 | 1 | complement(81885521..81946714) | 0.11 | -1.37 | -2.58 | 0.050 | 1.000 | 1.000 |
| Grin2a | 10 | 5930298..6119990 | 0.02 | -3.23 | -9.40 | 0.040 | 0.930 | 1.000 |
| Gse1 | 19 | 54060622..54148970 | 0.42 | -1.10 | -2.15 | 0.030 | 0.860 | 1.000 |
| Gsta1 | 9 | complement(27368272..27452902) | 669.05 | 1.11 | 2.15 | 0.030 | 0.860 | 1.000 |
| Gsta3 | 9 | 27402381..27417023 | 17.48 | 1.71 | 3.27 | 0.000 | 0.003 | 0.210 |
| Gsto2 | 1 | 267607416..267640455 | 0.37 | 2.54 | 5.80 | 0.002 | 0.250 | 1.000 |
| Gstt3 | 20 | 13817795..13825475 | 136.42 | 1.37 | 2.58 | 0.000 | 0.060 | 1.000 |
| Gtse1 | 7 | 126736732..126753220 | 0.81 | -2.17 | -4.51 | 0.000 | 0.004 | 0.270 |
| Gulo | 15 | complement(42671203..42693694) | 266.29 | 1.04 | 2.06 | 0.020 | 0.850 | 1.000 |
| Has3 | 19 | 39063998..39077722 | 0.04 | -1.70 | -3.26 | 0.030 | 0.860 | 1.000 |
| Haus1 | 18 | complement(74141481..74151950) | 0.30 | -2.13 | -4.37 | 0.030 | 0.870 | 1.000 |
| Haus4 | 15 | complement(33182128..33193537) | 1.04 | 1.48 | 2.79 | 0.004 | 0.380 | 1.000 |
| Hdc | 3 | complement(119057517..119075619) | 2.06 | 1.54 | 2.91 | 0.000 | 0.001 | 0.030 |
| Hes1 | 11 | complement(74312806..74315248) | 8.08 | -1.50 | -2.84 | 0.000 | 0.040 | 1.000 |
| Hhex | 1 | 256101903..256107649 | 86.58 | 2.13 | 4.38 | 0.000 | 0.000 | 0.000 |
| Hhip | 19 | 31524671..31614246 | 0.31 | -1.23 | -2.34 | 0.020 | 0.730 | 1.000 |
| Hipk2 | 4 | complement(66445266..66624912) | 1.88 | -1.67 | -3.18 | 0.000 | 0.050 | 1.000 |
| Hist1h1c | 17 | complement(43639749..43640387) | 31.10 | 1.05 | 2.08 | 0.000 | 0.020 | 1.000 |
| Hist1h1d | 17 | 43734461..43735120 | 0.28 | -3.61 | -12.21 | 0.002 | 0.260 | 1.000 |
| Hist1h2ah | 17 | 43632397..43632789 | 0.07 | -3.69 | -12.89 | 0.040 | 0.930 | 1.000 |
| Hist2h2ac | 2 | complement(198360301..198360678) | 0.11 | -6.13 | -69.94 | 0.010 | 0.700 | 1.000 |
| Hist2h4a | 17 | 43673294..43673605 | 0.36 | -4.37 | -20.63 | 0.002 | 0.270 | 1.000 |
| Hivep3 | 5 | 139007642..139077377 | 0.08 | -1.54 | -2.90 | 0.002 | 0.300 | 1.000 |
| Hmcn1 | 13 | complement(67799791..68360664) | 0.11 | -1.03 | -2.04 | 0.010 | 0.660 | 1.000 |
| Hmgb1 | 12 | 7081895..7094002 | 0.79 | 1.36 | 2.57 | 0.050 | 1.000 | 1.000 |
| Hmgn5b | 1 | 124477164..124485724 | 15.32 | 1.13 | 2.19 | 0.000 | 0.060 | 1.000 |
| Hprt1 | X | complement(158197149..158228749) | 2.04 | 1.61 | 3.06 | 0.010 | 0.710 | 1.000 |
| Hrasls | 11 | complement(75125952..75144903) | 1.36 | 3.89 | 14.87 | 0.000 | 0.000 | 0.000 |
| Hsd11b2 | 19 | 37476095..37481307 | 2.37 | 1.23 | 2.35 | 0.000 | 0.070 | 1.000 |
| Hsd17b2 | 19 | 50246402..50317891 | 112.92 | -1.51 | -2.84 | 0.000 | 0.001 | 0.050 |
| Hspa1b | 20 | complement(4877324..4879779) | 3.46 | -2.79 | -6.92 | 0.000 | 0.040 | 1.000 |
| Hspe1 | 9 | 61692154..61694599 | 169.29 | 1.16 | 2.23 | 0.000 | 0.070 | 1.000 |
| Htr2b | 9 | complement(93112805..93125014) | 0.04 | -4.70 | -25.99 | 0.003 | 0.370 | 1.000 |
| Igf1r | 1 | 128924966..129206516 | 0.03 | -2.10 | -4.28 | 0.030 | 0.880 | 1.000 |
| Igfbp1 | 14 | 87448692..87453785 | 25.76 | 1.11 | 2.16 | 0.002 | 0.240 | 1.000 |
| Igfbp5 | 9 | complement(80150211..80167033) | 0.18 | -1.07 | -2.11 | 0.030 | 0.920 | 1.000 |
| Igfbp6 | 7 | 143749221..143754054 | 1.12 | -1.39 | -2.62 | 0.004 | 0.390 | 1.000 |
| Il17re | 4 | 145413230..145426603 | 0.14 | -1.55 | -2.93 | 0.020 | 0.830 | 1.000 |
| Il33 | 1 | 248132090..248147029 | 31.10 | 1.11 | 2.17 | 0.000 | 0.020 | 1.000 |
| Inmt | 4 | complement(85381889..85386231) | 34.16 | 1.58 | 2.99 | 0.002 | 0.270 | 1.000 |
| Ino80d | 9 | complement(69830424..69878706) | 0.49 | -1.56 | -2.94 | 0.003 | 0.330 | 1.000 |
| Irgq | 1 | 81395841..81399672 | 0.94 | -1.03 | -2.04 | 0.020 | 0.840 | 1.000 |
| Irx1 | 1 | 33910912..33916741 | 3.90 | 1.40 | 2.64 | 0.000 | 0.005 | 0.340 |
| Isyna1 | 16 | complement(20589481..20592449) | 4.73 | -1.03 | -2.04 | 0.002 | 0.230 | 1.000 |
| Itpr2 | 4 | complement(180538389..180722358) | 1.84 | -1.03 | -2.04 | 0.003 | 0.310 | 1.000 |
| Jakmip2 | 18 | complement(37869289..38088457) | 0.11 | 2.47 | 5.54 | 0.008 | 0.550 | 1.000 |
| Jun | 5 | complement(114011189..114014277) | 12.36 | 1.05 | 2.06 | 0.007 | 0.540 | 1.000 |
| Kbtbd6_1 | 15 | 61692116..61693911 | 0.41 | -1.06 | -2.08 | 0.030 | 0.870 | 1.000 |
| Kcna2 | 2 | 209838869..209852471 | 0.16 | 1.07 | 2.09 | 0.030 | 0.860 | 1.000 |
| Kcnab1 | 2 | 155718359..156009526 | 0.09 | -1.49 | -2.80 | 0.040 | 0.970 | 1.000 |
| Kcnh1 | 13 | 110920737..111232269 | 0.21 | -1.96 | -3.90 | 0.002 | 0.310 | 1.000 |
| Kcnk2 | 13 | complement(107690087..107886476) | 0.03 | -1.94 | -3.83 | 0.050 | 1.000 | 1.000 |
| Kcnk3 | 6 | complement(27151612..27190132) | 0.15 | -1.07 | -2.10 | 0.040 | 0.990 | 1.000 |
| Kcnn2_2 | 18 | 39335377..39479257 | 0.50 | -3.18 | -9.06 | 0.000 | 0.009 | 0.710 |
| Kcns3 | 6 | complement(36764044..36819821) | 0.11 | 1.90 | 3.73 | 0.050 | 1.000 | 1.000 |
| Kctd7 | 12 | complement(30024081..30033357) | 0.90 | -1.02 | -2.03 | 0.020 | 0.850 | 1.000 |
| Kif20a | 18 | 27424328..27432814 | 0.42 | -1.09 | -2.12 | 0.020 | 0.830 | 1.000 |
| Kif23 | 8 | complement(66866082..66893196) | 0.10 | -1.65 | -3.13 | 0.030 | 0.910 | 1.000 |
| Kif2c | 5 | complement(136027934..136053210) | 0.17 | -1.25 | -2.38 | 0.050 | 1.000 | 1.000 |
| Kif5a | 7 | complement(70513343..70556827) | 0.09 | -1.16 | -2.23 | 0.040 | 0.950 | 1.000 |
| Kmt2a | 8 | complement(49114990..49158971) | 0.96 | -1.19 | -2.28 | 0.002 | 0.280 | 1.000 |
| Knop1_1 | 1 | complement(188368581..188373571) | 0.16 | -6.56 | -94.38 | 0.005 | 0.440 | 1.000 |
| Knstrn | 3 | 110618298..110638046 | 0.24 | -1.79 | -3.46 | 0.030 | 0.910 | 1.000 |
| Krt2 | 7 | complement(143412858..143420027) | 0.88 | -1.32 | -2.50 | 0.008 | 0.560 | 1.000 |
| Ksr2 | 12 | complement(44536293..44911147) | 0.93 | -1.01 | -2.01 | 0.030 | 0.910 | 1.000 |
| L3hypdh | 6 | complement(94824112..94834908) | 4.05 | 1.17 | 2.25 | 0.000 | 0.030 | 1.000 |
| Lama3 | 18 | 3705916..3940838 | 1.37 | 1.50 | 2.83 | 0.000 | 0.000 | 0.009 |
| Lama5 | 3 | complement(175553045..175601127) | 0.08 | -1.02 | -2.03 | 0.040 | 0.970 | 1.000 |
| Lilrb3l | 1 | 64074231..64081438 | 0.66 | -1.19 | -2.29 | 0.004 | 0.400 | 1.000 |
| Lingo4 | 2 | 195617044..195643502 | 0.35 | 2.64 | 6.24 | 0.000 | 0.000 | 0.002 |
| LOC100359600 | 6 | 10642700..10644676 | 2.44 | -1.49 | -2.81 | 0.000 | 0.001 | 0.040 |
| LOC100359951 | 9 | complement(1012091..1012450) | 0.95 | 2.65 | 6.28 | 0.030 | 0.860 | 1.000 |
| LOC100360117 | 7 | 117967818..117969850 | 0.17 | -5.07 | -33.56 | 0.030 | 0.910 | 1.000 |
| LOC100360781 | 9 | 69790831..69791196 | 3.98 | 1.10 | 2.15 | 0.030 | 0.910 | 1.000 |
| LOC100361087 | 13 | complement(88636875..88943592) | 1.96 | -1.56 | -2.96 | 0.001 | 0.130 | 1.000 |
| LOC100361457 | 10 | complement(109519134..109520846) | 0.14 | -8.09 | -271.73 | 0.001 | 0.160 | 1.000 |
| LOC100361547_2 | 1 | 148240504..148287217 | 1.16 | 1.16 | 2.24 | 0.020 | 0.730 | 1.000 |
| LOC100362027 | 7 | complement(73213581..73216251) | 35.85 | 4.79 | 27.72 | 0.000 | 0.010 | 1.000 |
| LOC100362339 | 17 | 54057964..54058460 | 0.08 | -4.48 | -22.30 | 0.010 | 0.650 | 1.000 |
| LOC100363193 | X | 54266687..54295766 | 0.20 | -2.54 | -5.82 | 0.001 | 0.200 | 1.000 |
| LOC100364769 | 1 | 217699180..217735103 | 0.18 | -5.68 | -51.13 | 0.000 | 0.020 | 1.000 |
| LOC100909609 | 1 | 206527987..206573249 | 0.01 | -4.68 | -25.72 | 0.008 | 0.550 | 1.000 |
| LOC100909709 | 8 | complement(103503985..103554468) | 0.15 | -1.64 | -3.11 | 0.006 | 0.500 | 1.000 |
| LOC100909784_1 | 4 | 51553454..51561174 | 0.12 | 3.18 | 9.04 | 0.020 | 0.760 | 1.000 |
| LOC100909879 | 2 | 87418517..87419861 | 0.43 | 1.72 | 3.30 | 0.040 | 1.000 | 1.000 |
| LOC100910021 | 8 | complement(106863494..106870994) | 0.11 | -6.24 | -75.48 | 0.009 | 0.570 | 1.000 |
| LOC100910438 | 2 | 28370373..28381311 | 0.34 | 7.29 | 156.53 | 0.010 | 0.610 | 1.000 |
| LOC100910807 | X | 124321551..124323960 | 0.21 | -6.91 | -120.66 | 0.002 | 0.270 | 1.000 |
| LOC100910979_2 | 18 | 55576239..55584957 | 33.33 | 2.36 | 5.13 | 0.000 | 0.080 | 1.000 |
| LOC100911027 | 1 | complement(54719971..54748763) | 0.11 | -4.95 | -30.83 | 0.030 | 0.910 | 1.000 |
| LOC100911256 | 6 | complement(91207178..91250138) | 0.05 | -2.24 | -4.73 | 0.005 | 0.450 | 1.000 |
| LOC100911337_3 | 4 | complement(120413653..120414118) | 1.55 | -4.43 | -21.60 | 0.000 | 0.030 | 1.000 |
| LOC100911365 | 1 | complement(214389255..214394411) | 0.23 | -3.56 | -11.82 | 0.006 | 0.510 | 1.000 |
| LOC100911374 | 11 | complement(72786730..72814850) | 0.32 | -6.40 | -84.65 | 0.006 | 0.490 | 1.000 |
| LOC100911545 | 4 | 154423209..154472924 | 0.29 | -8.71 | -419.14 | 0.000 | 0.040 | 1.000 |
| LOC100911564 | 9 | 16314173..16317413 | 0.08 | -2.76 | -6.78 | 0.050 | 1.000 | 1.000 |
| LOC100911672 | 4 | complement(157267901..157274755) | 0.55 | -1.98 | -3.96 | 0.005 | 0.440 | 1.000 |
| LOC100911807 | 8 | complement(118428289..118436894) | 0.03 | -6.47 | -88.41 | 0.009 | 0.590 | 1.000 |
| LOC100911881 | 1 | complement(214039481..214074663) | 0.24 | -3.80 | -13.89 | 0.003 | 0.350 | 1.000 |
| LOC100912026 | 8 | complement(38545715..38549268) | 166.75 | 1.34 | 2.53 | 0.000 | 0.080 | 1.000 |
| LOC100912489 | 2 | complement(198411955..198412350) | 0.08 | -5.68 | -51.37 | 0.020 | 0.830 | 1.000 |
| LOC102549115 | 1 | 88451958..88453578 | 0.34 | -2.50 | -5.67 | 0.008 | 0.550 | 1.000 |
| LOC102549542 | 2 | 234375315..234483149 | 3.33 | 1.23 | 2.35 | 0.001 | 0.130 | 1.000 |
| LOC102550314 | 15 | 5808006..5809699 | 1.92 | 9.35 | 654.48 | 0.001 | 0.210 | 1.000 |
| LOC102552055 | 5 | complement(165312130..165316652) | 0.06 | -3.31 | -9.94 | 0.010 | 0.620 | 1.000 |
| LOC103689940 | 2 | complement(216339154..216382260) | 0.16 | 2.04 | 4.10 | 0.009 | 0.570 | 1.000 |
| LOC103689941 | 5 | 142303636..142330854 | 0.05 | -4.79 | -27.59 | 0.050 | 1.000 | 1.000 |
| LOC103689943 | 10 | 28243..72912 | 0.25 | -1.40 | -2.64 | 0.001 | 0.220 | 1.000 |
| LOC103689993 | X | complement(45244038..45259492) | 0.32 | -3.77 | -13.66 | 0.001 | 0.220 | 1.000 |
| LOC103690002 | 4 | 170149029..170149418 | 0.09 | -5.92 | -60.67 | 0.020 | 0.770 | 1.000 |
| LOC103690016 | 1 | 197659187..197681006 | 0.62 | -2.97 | -7.81 | 0.001 | 0.190 | 1.000 |
| LOC103690114 | 1 | 87790104..87801872 | 0.04 | -4.79 | -27.59 | 0.050 | 1.000 | 1.000 |
| LOC103690120 | 4 | complement(117767077..117767772) | 0.51 | 4.21 | 18.50 | 0.003 | 0.350 | 1.000 |
| LOC103692170 | 4 | 113935492..113941478 | 0.84 | -1.98 | -3.94 | 0.030 | 0.860 | 1.000 |
| LOC103692976 | 7 | complement(140154490..140172448) | 0.54 | -1.20 | -2.30 | 0.009 | 0.590 | 1.000 |
| LOC103693776 | 14 | 86514214..86530701 | 5.31 | -1.05 | -2.08 | 0.001 | 0.220 | 1.000 |
| LOC108348050 | 11 | 15081774..15089850 | 0.30 | 5.70 | 51.87 | 0.050 | 1.000 | 1.000 |
| LOC108348055 | 10 | 49020454..49028069 | 1.61 | 1.25 | 2.37 | 0.020 | 0.780 | 1.000 |
| LOC108348082 | 10 | 47182433..47203753 | 6.57 | 2.34 | 5.05 | 0.000 | 0.060 | 1.000 |
| LOC108348118 | 1 | 68436593..68488427 | 0.34 | -1.25 | -2.37 | 0.020 | 0.830 | 1.000 |
| LOC108348142 | 7 | 118507224..118509256 | 0.22 | -5.35 | -40.84 | 0.020 | 0.830 | 1.000 |
| LOC108348155 | 12 | complement(21356475..21362205) | 0.04 | -4.82 | -28.31 | 0.040 | 0.970 | 1.000 |
| LOC108348189 | 7 | 117963740..117964414 | 0.36 | -2.37 | -5.18 | 0.030 | 0.920 | 1.000 |
| LOC108348215 | 1 | 61445672..61509765 | 0.65 | 1.57 | 2.97 | 0.000 | 0.090 | 1.000 |
| LOC257642_1 | 6 | complement(30638738..30643594) | 2.55 | -8.00 | -256.77 | 0.000 | 0.000 | 0.002 |
| LOC257642_2 | 14 | complement(46523594..46529375) | 6313.70 | -7.59 | -192.08 | 0.000 | 0.000 | 0.000 |
| LOC257642_3 | 14 | complement(46588411..46593828) | 2.25 | -7.82 | -226.70 | 0.000 | 0.000 | 0.003 |
| LOC257642_4 | 14 | complement(46653040..46657975) | 5679.99 | -7.62 | -197.28 | 0.000 | 0.000 | 0.000 |
| LOC317456 | X | 43881246..43882204 | 0.26 | -8.10 | -273.53 | 0.001 | 0.130 | 1.000 |
| LOC367746 | X | 18163358..18164065 | 5.25 | -1.95 | -3.87 | 0.000 | 0.005 | 0.370 |
| LOC499407 | 20 | 7330250..7361559 | 0.15 | -1.79 | -3.46 | 0.008 | 0.560 | 1.000 |
| LOC499823 | 3 | 71437241..71450807 | 0.15 | -7.27 | -154.23 | 0.003 | 0.310 | 1.000 |
| LOC500035 | 4 | 40161285..40169564 | 0.24 | -8.09 | -271.73 | 0.001 | 0.160 | 1.000 |
| LOC501297 | 19 | complement(340946..345698) | 1.03 | -2.44 | -5.44 | 0.001 | 0.160 | 1.000 |
| LOC682206 | 1 | 61733805..61740311 | 1.37 | 1.05 | 2.07 | 0.007 | 0.510 | 1.000 |
| LOC686087 | X | 157759624..157773108 | 3.17 | 1.55 | 2.93 | 0.001 | 0.220 | 1.000 |
| LOC689065 | 1 | complement(219743747..219745654) | 0.57 | -1.24 | -2.37 | 0.030 | 0.880 | 1.000 |
| LOC689986 | 14 | complement(3248080..3288017) | 0.08 | 3.90 | 14.92 | 0.009 | 0.590 | 1.000 |
| LOC691716 | 1 | 196095214..196095597 | 0.44 | 2.36 | 5.13 | 0.050 | 1.000 | 1.000 |
| Lonrf3 | X | 122938009..122973321 | 0.70 | 2.13 | 4.38 | 0.000 | 0.000 | 0.020 |
| Loxl4 | 1 | complement(261835992..261853495) | 2.87 | -1.00 | -2.00 | 0.010 | 0.650 | 1.000 |
| Lpl | 16 | complement(22537056..22561496) | 0.80 | -1.21 | -2.31 | 0.002 | 0.280 | 1.000 |
| Lpp | 11 | complement(79205730..79703736) | 0.55 | -1.62 | -3.08 | 0.003 | 0.350 | 1.000 |
| Lrfn4 | 1 | complement(219850037..219853329) | 0.67 | -1.59 | -3.01 | 0.000 | 0.080 | 1.000 |
| Lrrc4 | 4 | complement(56110658..56114254) | 0.04 | -2.17 | -4.50 | 0.020 | 0.840 | 1.000 |
| Lrrn4 | 3 | complement(125523781..125533600) | 0.20 | -1.92 | -3.78 | 0.010 | 0.650 | 1.000 |
| Lypd3 | 1 | 81499821..81504318 | 0.19 | -1.43 | -2.69 | 0.040 | 1.000 | 1.000 |
| Map2k6 | 10 | 98706960..98823287 | 3.92 | 2.27 | 4.82 | 0.000 | 0.000 | 0.000 |
| Mapk10 | 14 | 8080275..8368254 | 0.09 | -3.66 | -12.61 | 0.000 | 0.060 | 1.000 |
| Marveld1 | 1 | 261389804..261393736 | 10.39 | -1.08 | -2.11 | 0.020 | 0.770 | 1.000 |
| Mast4 | 2 | complement(32444565..32518643) | 0.09 | -8.67 | -406.03 | 0.000 | 0.040 | 1.000 |
| Mcm6 | 13 | complement(45042882..45068077) | 0.97 | 1.47 | 2.76 | 0.003 | 0.370 | 1.000 |
| Mcrip2 | 10 | complement(15230801..15235740) | 9.42 | 1.35 | 2.54 | 0.000 | 0.030 | 1.000 |
| Mepce | 12 | 21721837..21725435 | 0.02 | -6.31 | -79.19 | 0.010 | 0.650 | 1.000 |
| Mex3b | 1 | 144831523..144835747 | 0.29 | -1.57 | -2.98 | 0.001 | 0.220 | 1.000 |
| MGC114492 | X | complement(139260135..139263974) | 0.32 | 1.59 | 3.01 | 0.030 | 0.890 | 1.000 |
| MGC94199_2 | 5 | 24146171..24165463 | 0.47 | -2.03 | -4.08 | 0.040 | 0.950 | 1.000 |
| Mipol1 | 6 | 78172790..78499599 | 0.49 | 1.18 | 2.27 | 0.030 | 0.860 | 1.000 |
| Mme | 2 | 153803349..153880738 | 0.55 | 2.09 | 4.25 | 0.001 | 0.110 | 1.000 |
| Mocs2 | 2 | 46980976..46992883 | 58.11 | 1.33 | 2.52 | 0.000 | 0.005 | 0.360 |
| Mospd1 | 14 | complement(87699494..87701884) | 7.50 | 1.04 | 2.06 | 0.001 | 0.130 | 1.000 |
| Mroh9 | 13 | complement(80928995..80968971) | 0.16 | 2.83 | 7.13 | 0.003 | 0.310 | 1.000 |
| Ms4a18 | 1 | complement(227051289..227067915) | 0.08 | -4.61 | -24.50 | 0.050 | 1.000 | 1.000 |
| Msln | 10 | complement(15119663..15125408) | 0.94 | -2.37 | -5.18 | 0.000 | 0.010 | 1.000 |
| Mt1 | 17 | 78793336..78793724 | 124.11 | -2.39 | -5.23 | 0.000 | 0.000 | 0.010 |
| Mt2A | 19 | complement(11307967..11308740) | 49.76 | -2.22 | -4.67 | 0.000 | 0.000 | 0.020 |
| Mthfr | 5 | 164845925..164860910 | 1.73 | -1.10 | -2.14 | 0.007 | 0.540 | 1.000 |
| Muc16 | 8 | complement(18437787..18508971) | 0.12 | -2.25 | -4.77 | 0.001 | 0.130 | 1.000 |
| Muc6 | 1 | complement(214608704..214629938) | 0.03 | -4.12 | -17.39 | 0.000 | 0.060 | 1.000 |
| Mybl1 | 5 | 9279970..9315114 | 0.93 | -2.28 | -4.85 | 0.000 | 0.000 | 0.010 |
| Myc | 7 | 102586313..102591240 | 5.25 | 2.13 | 4.37 | 0.000 | 0.000 | 0.000 |
| Mzf1 | 1 | 65522118..65533212 | 0.19 | -1.21 | -2.31 | 0.020 | 0.780 | 1.000 |
| N5 | 4 | complement(57588724..57590923) | 0.05 | -4.89 | -29.74 | 0.001 | 0.130 | 1.000 |
| Nat2 | 16 | complement(23961067..23991570) | 1.67 | 1.17 | 2.25 | 0.010 | 0.680 | 1.000 |
| Nat8f3 | 4 | complement(117490035..117490721) | 2.29 | 3.01 | 8.08 | 0.000 | 0.000 | 0.005 |
| Nat8f5 | 4 | complement(117506732..117531480) | 0.36 | 1.92 | 3.80 | 0.020 | 0.810 | 1.000 |
| Nav1 | 13 | complement(52267254..52514875) | 0.20 | -1.24 | -2.36 | 0.007 | 0.540 | 1.000 |
| Nav3 | 7 | complement(52165233..52404774) | 0.24 | -1.04 | -2.05 | 0.040 | 0.950 | 1.000 |
| Ndufc1 | 2 | complement(140462900..140464607) | 101.24 | 1.03 | 2.04 | 0.001 | 0.190 | 1.000 |
| Net1 | 17 | 70010794..70020982 | 23.96 | 1.07 | 2.09 | 0.000 | 0.040 | 1.000 |
| Neu2 | 9 | 94702129..94724902 | 0.81 | 2.32 | 5.00 | 0.000 | 0.005 | 0.410 |
| NEWGENE_1592020 | 2 | complement(185289653..185303610) | 0.02 | -5.39 | -42.02 | 0.030 | 0.890 | 1.000 |
| NEWGENE_2319083 | 1 | complement(13302256..13341952) | 1.37 | 1.01 | 2.01 | 0.010 | 0.680 | 1.000 |
| Nfatc2 | 3 | complement(165245238..165360292) | 0.23 | -1.46 | -2.75 | 0.008 | 0.570 | 1.000 |
| Nfe2l3_2 | 4 | 81205404..81232813 | 0.02 | -6.13 | -69.94 | 0.010 | 0.700 | 1.000 |
| Nfil3 | 17 | 12261102..12276315 | 21.38 | 1.04 | 2.06 | 0.000 | 0.040 | 1.000 |
| Ngef | 9 | complement(94569286..94601852) | 9.17 | 1.16 | 2.23 | 0.000 | 0.003 | 0.200 |
| Nilr1 | 1 | 63734135..63741982 | 0.13 | -1.81 | -3.52 | 0.020 | 0.730 | 1.000 |
| Nim1k | 2 | complement(52448715..52492509) | 0.80 | -1.41 | -2.67 | 0.050 | 1.000 | 1.000 |
| Ninj2 | 4 | 152630469..152733633 | 0.58 | 1.35 | 2.55 | 0.020 | 0.780 | 1.000 |
| Nipal1 | 14 | complement(38104047..38128024) | 7.04 | 1.01 | 2.01 | 0.001 | 0.120 | 1.000 |
| Nmrk1 | 1 | complement(234721974..234749447) | 3.16 | 1.38 | 2.60 | 0.001 | 0.130 | 1.000 |
| Npas2 | 9 | 45901741..46081880 | 2.67 | -1.43 | -2.69 | 0.000 | 0.070 | 1.000 |
| Nptx2 | 12 | complement(12013911..12025549) | 0.52 | 1.32 | 2.50 | 0.004 | 0.390 | 1.000 |
| Nr0b2 | 5 | 151776004..151779319 | 23.78 | -2.18 | -4.52 | 0.000 | 0.003 | 0.230 |
| Nr1d1 | 10 | complement(86683875..86690815) | 14.37 | -5.05 | -33.13 | 0.000 | 0.000 | 0.000 |
| Nr1d2 | 15 | 8730871..8757165 | 6.28 | -1.19 | -2.29 | 0.001 | 0.120 | 1.000 |
| Nrcam | 6 | 64297888..64864996 | 0.16 | -1.19 | -2.28 | 0.010 | 0.650 | 1.000 |
| Nrg4 | 8 | complement(59782974..59849490) | 6.10 | 2.00 | 3.99 | 0.000 | 0.020 | 1.000 |
| Nrxn1 | 6 | complement(13886740..15191660) | 0.05 | -1.65 | -3.14 | 0.020 | 0.830 | 1.000 |
| Ntng1 | 2 | complement(212696624..212852870) | 0.29 | -1.05 | -2.07 | 0.010 | 0.710 | 1.000 |
| Nudt11 | X | 17540458..17540952 | 0.53 | -3.26 | -9.59 | 0.003 | 0.360 | 1.000 |
| Nynrin | 15 | 34520142..34539930 | 0.12 | -1.16 | -2.24 | 0.030 | 0.860 | 1.000 |
| Oas1i | 12 | 42343123..42355131 | 0.24 | 6.71 | 104.71 | 0.020 | 0.820 | 1.000 |
| Oat | 1 | complement(204562289..204582070) | 161.12 | 1.13 | 2.19 | 0.002 | 0.250 | 1.000 |
| Obp1f_2 | X | complement(83847944..83864150) | 1.31 | 1.07 | 2.10 | 0.040 | 1.000 | 1.000 |
| Odf3b | 7 | complement(130347945..130350570) | 2.17 | -2.23 | -4.71 | 0.000 | 0.020 | 1.000 |
| Ogdhl | 16 | 8497569..8523557 | 0.15 | -1.32 | -2.50 | 0.040 | 0.980 | 1.000 |
| Olr1353 | 9 | 99795678..99796732 | 2.80 | -1.52 | -2.86 | 0.002 | 0.240 | 1.000 |
| Onecut1 | 8 | 81766041..81793577 | 10.87 | 1.81 | 3.51 | 0.000 | 0.070 | 1.000 |
| Onecut2 | 18 | 59830363..59875127 | 4.13 | -1.07 | -2.10 | 0.003 | 0.310 | 1.000 |
| Otogl | 7 | 51794173..51935142 | 0.12 | -2.46 | -5.50 | 0.001 | 0.160 | 1.000 |
| Pcdh10 | 2 | 136993208..137014492 | 0.19 | -2.74 | -6.70 | 0.000 | 0.060 | 1.000 |
| Pcdhga4 | 18 | 30831365..30833782 | 0.02 | -2.86 | -7.28 | 0.030 | 0.880 | 1.000 |
| Pcdhgb4 | 18 | 30869628..30872006 | 0.05 | -2.14 | -4.40 | 0.030 | 0.910 | 1.000 |
| Pde11a | 3 | complement(62818502..63211845) | 0.08 | -2.54 | -5.80 | 0.003 | 0.360 | 1.000 |
| Pde1b | 7 | 145117951..145145376 | 0.06 | -4.86 | -28.97 | 0.030 | 0.920 | 1.000 |
| Pde3a | 4 | 175431904..175700238 | 0.18 | -1.46 | -2.75 | 0.008 | 0.550 | 1.000 |
| Pdzd2 | 2 | complement(62399748..62634785) | 0.14 | -1.18 | -2.27 | 0.020 | 0.780 | 1.000 |
| Pitx2 | 2 | 233602732..233621129 | 0.03 | -3.05 | -8.26 | 0.030 | 0.910 | 1.000 |
| Pla2g2a | 5 | 157282669..157285328 | 1.61 | -1.00 | -2.00 | 0.030 | 0.910 | 1.000 |
| Pla2g4c | 1 | 75428027..75620911 | 0.14 | -1.61 | -3.06 | 0.010 | 0.700 | 1.000 |
| Plce1 | 1 | 257157264..257466064 | 1.45 | -1.13 | -2.20 | 0.000 | 0.010 | 0.940 |
| Plec | 7 | complement(117230319..117289961) | 1.87 | -1.04 | -2.05 | 0.002 | 0.270 | 1.000 |
| Plxna4 | 4 | complement(59445767..59809321) | 0.19 | -2.98 | -7.89 | 0.000 | 0.000 | 0.004 |
| Pnck | X | 157150655..157154555 | 0.18 | -2.88 | -7.37 | 0.001 | 0.220 | 1.000 |
| Polg | 1 | complement(141172531..141188031) | 14.74 | -1.26 | -2.39 | 0.000 | 0.030 | 1.000 |
| Popdc2 | 11 | complement(64936692..64952687) | 0.21 | -1.32 | -2.50 | 0.030 | 0.860 | 1.000 |
| Pou2f1 | 13 | complement(84083632..84217366) | 0.21 | -1.42 | -2.68 | 0.020 | 0.790 | 1.000 |
| Ppara | 7 | 126619196..126681752 | 6.52 | -1.13 | -2.19 | 0.001 | 0.220 | 1.000 |
| Ppp1r9a | 4 | 29978739..30241911 | 0.06 | -2.17 | -4.50 | 0.010 | 0.700 | 1.000 |
| Ppp2r2b | 18 | complement(36985714..37245809) | 0.26 | -2.10 | -4.29 | 0.002 | 0.260 | 1.000 |
| Ppp2r3b | 14 | 1469748..1476355 | 0.60 | -1.02 | -2.03 | 0.010 | 0.680 | 1.000 |
| Prkcdbp | 1 | complement(170317113..170318935) | 44.50 | -1.08 | -2.11 | 0.000 | 0.040 | 1.000 |
| Prmt6 | 2 | complement(213420314..213421811) | 0.14 | 5.64 | 49.89 | 0.040 | 1.000 | 1.000 |
| Prnp | 3 | 124515978..124531316 | 1.40 | -1.09 | -2.12 | 0.005 | 0.480 | 1.000 |
| Pttg1 | 10 | complement(29020049..29026002) | 0.33 | -1.62 | -3.07 | 0.020 | 0.850 | 1.000 |
| Rad9b | 12 | 39699181..39729516 | 0.53 | 1.24 | 2.36 | 0.030 | 0.870 | 1.000 |
| Rasl10b | 10 | 70627401..70637332 | 0.60 | 1.62 | 3.08 | 0.003 | 0.360 | 1.000 |
| Rbm12_1 | 3 | complement(151616037..151625644) | 1.20 | -1.62 | -3.08 | 0.020 | 0.840 | 1.000 |
| Rcan2 | 9 | complement(19518558..19749145) | 3.51 | 1.13 | 2.20 | 0.000 | 0.070 | 1.000 |
| Rcor3 | 13 | complement(110822150..110864469) | 0.64 | -1.04 | -2.06 | 0.020 | 0.790 | 1.000 |
| Rec114 | 8 | complement(63445644..63533546) | 0.84 | 2.28 | 4.84 | 0.006 | 0.510 | 1.000 |
| Reln | 4 | 9347528..9773670 | 1.00 | -1.10 | -2.14 | 0.001 | 0.220 | 1.000 |
| Resp18 | 9 | complement(82470759..82477181) | 0.26 | -2.21 | -4.63 | 0.009 | 0.590 | 1.000 |
| RGD1304963 | 6 | 23337571..23346173 | 0.08 | 2.58 | 5.99 | 0.008 | 0.550 | 1.000 |
| RGD1305184 | 18 | 55666027..55667668 | 0.16 | 3.21 | 9.26 | 0.020 | 0.770 | 1.000 |
| RGD1306626 | 4 | complement(27606078..27638676) | 0.28 | 1.55 | 2.94 | 0.050 | 1.000 | 1.000 |
| RGD1309362 | 18 | 55505993..55508704 | 3.83 | 2.08 | 4.21 | 0.020 | 0.840 | 1.000 |
| RGD1309534 | 8 | complement(13838572..13861212) | 52.20 | 1.43 | 2.69 | 0.000 | 0.003 | 0.200 |
| RGD1559459 | 14 | 22937421..22952822 | 3.10 | 3.18 | 9.08 | 0.000 | 0.000 | 0.000 |
| RGD1559588_2 | 12 | 19890749..19894166 | 0.03 | -5.03 | -32.57 | 0.040 | 1.000 | 1.000 |
| RGD1559724 | 6 | complement(75469895..75470347) | 4.67 | 1.14 | 2.20 | 0.009 | 0.590 | 1.000 |
| RGD1560925 | 9 | complement(105531195..105655471) | 0.06 | -2.04 | -4.11 | 0.020 | 0.770 | 1.000 |
| RGD1561662 | 9 | complement(15546823..15582556) | 2.48 | -1.00 | -2.01 | 0.007 | 0.530 | 1.000 |
| RGD1561849 | 5 | complement(9355507..9381214) | 2.88 | -2.07 | -4.20 | 0.000 | 0.000 | 0.020 |
| RGD1563072 | 5 | 144634143..144634823 | 0.17 | -2.73 | -6.63 | 0.020 | 0.840 | 1.000 |
| RGD1564698 | 10 | complement(19715275..19715832) | 2.87 | 1.02 | 2.03 | 0.030 | 0.910 | 1.000 |
| RGD1564865 | 17 | 69468427..69497132 | 31.14 | 1.40 | 2.64 | 0.000 | 0.040 | 1.000 |
| RGD1565785 | X | 54390733..54409466 | 0.24 | 1.61 | 3.06 | 0.040 | 0.990 | 1.000 |
| Rgs18 | 13 | complement(61565483..61591139) | 1.99 | 1.18 | 2.26 | 0.020 | 0.770 | 1.000 |
| Rhof | 12 | 38880377..38894582 | 1.69 | -1.66 | -3.16 | 0.000 | 0.000 | 0.003 |
| Rn50_10_0698.6 | 10 | 70100661..70101169 | 26.13 | 1.16 | 2.24 | 0.000 | 0.040 | 1.000 |
| Rn50_13_0828.1 | 13 | 77784808..77822333 | 5.71 | 1.02 | 2.03 | 0.000 | 0.040 | 1.000 |
| Rn50_20_0041.1 | 20 | 1980610..1983147 | 0.33 | -1.69 | -3.22 | 0.040 | 0.970 | 1.000 |
| Rn50_5_0791.1 | 5 | 74727494..74954576 | 0.62 | -1.44 | -2.71 | 0.003 | 0.330 | 1.000 |
| Rn60_1_2212.3 | 1 | complement(221151827..221153278) | 15.28 | -5.07 | -33.70 | 0.000 | 0.000 | 0.000 |
| Rn60_12_0160.2 | 12 | 15926171..15927570 | 0.05 | -4.29 | -19.63 | 0.010 | 0.640 | 1.000 |
| Rn60_16_0592.1 | 16 | 59116709..59124402 | 0.16 | -2.60 | -6.07 | 0.030 | 0.870 | 1.000 |
| Rn60_3_0143.1 | 3 | 14192061..14202271 | 0.13 | -6.13 | -69.94 | 0.010 | 0.700 | 1.000 |
| Rn60_5_1207.2 | 5 | 120498890..120682283 | 0.03 | -4.94 | -30.66 | 0.040 | 0.970 | 1.000 |
| Robo1 | 11 | 9642365..10143317 | 0.15 | -1.22 | -2.33 | 0.020 | 0.730 | 1.000 |
| Rorc | 2 | 195617021..195637630 | 40.03 | 1.75 | 3.36 | 0.000 | 0.000 | 0.000 |
| Rpl13a_2 | 1 | complement(101120711..101131193) | 0.44 | 2.77 | 6.83 | 0.008 | 0.550 | 1.000 |
| Rpl35al1 | 6 | 135139292..135139621 | 3.99 | -10.50 | -1450.78 | 0.000 | 0.003 | 0.190 |
| Rps18l1 | 10 | complement(104521700..104522241) | 18.09 | 1.29 | 2.44 | 0.040 | 0.970 | 1.000 |
| Rsph9 | 9 | 17225122..17245093 | 1.10 | 1.12 | 2.17 | 0.010 | 0.700 | 1.000 |
| Ryr2 | 17 | complement(65535403..65955606) | 0.03 | -1.41 | -2.66 | 0.050 | 1.000 | 1.000 |
| Sall4 | 3 | complement(165520392..165537940) | 0.10 | -2.63 | -6.18 | 0.005 | 0.440 | 1.000 |
| Samd4a | 15 | 23665202..23878539 | 1.86 | -1.43 | -2.70 | 0.000 | 0.002 | 0.130 |
| Scn8a | 7 | 142575672..142684114 | 0.05 | -2.22 | -4.65 | 0.004 | 0.420 | 1.000 |
| Sdhaf3 | 4 | 32495321..32558798 | 7.71 | 1.01 | 2.01 | 0.006 | 0.480 | 1.000 |
| Semg1 | 3 | complement(160627610..160630457) | 0.15 | -3.20 | -9.16 | 0.020 | 0.790 | 1.000 |
| Serpina7 | X | complement(110226572..110232179) | 0.64 | -1.54 | -2.91 | 0.010 | 0.610 | 1.000 |
| Setd1b | 12 | complement(38845621..38869346) | 2.06 | -1.54 | -2.91 | 0.000 | 0.010 | 0.750 |
| Sidt1 | 11 | 61321459..61417842 | 0.16 | -1.38 | -2.61 | 0.020 | 0.780 | 1.000 |
| Slc1a2 | 3 | 92640752..92665644 | 4.88 | 1.37 | 2.58 | 0.000 | 0.040 | 1.000 |
| Slc1a4 | 14 | complement(104581190..104612597) | 0.48 | -1.27 | -2.41 | 0.008 | 0.550 | 1.000 |
| Slc22a3 | 1 | 48433079..48521256 | 0.06 | 2.68 | 6.40 | 0.030 | 0.870 | 1.000 |
| Slc24a1 | 8 | complement(70408528..70436028) | 0.01 | -4.70 | -25.94 | 0.040 | 0.960 | 1.000 |
| Slc25a21 | 6 | complement(77624385..77848434) | 2.44 | 1.21 | 2.31 | 0.000 | 0.070 | 1.000 |
| Slc25a30 | 15 | complement(57813356..57834173) | 5.47 | 1.02 | 2.02 | 0.006 | 0.490 | 1.000 |
| Slc25a47 | 6 | 132762280..132768379 | 95.82 | -1.43 | -2.70 | 0.000 | 0.005 | 0.360 |
| Slc27a5 | 1 | 65576535..65587186 | 202.54 | 1.00 | 2.00 | 0.030 | 0.880 | 1.000 |
| Slc2a10 | 3 | 162181974..162194804 | 0.03 | -2.26 | -4.80 | 0.030 | 0.890 | 1.000 |
| Slc35d2 | 17 | complement(1618189..1648973) | 9.97 | -1.04 | -2.05 | 0.000 | 0.030 | 1.000 |
| Slc40a1_1 | 9 | complement(52820683..52830457) | 0.03 | -6.13 | -69.94 | 0.010 | 0.700 | 1.000 |
| Slc44a3 | 2 | complement(225038582..225107283) | 0.21 | 1.71 | 3.26 | 0.050 | 1.000 | 1.000 |
| Slc6a6 | 4 | complement(123642210..123713464) | 1.86 | -2.76 | -6.75 | 0.000 | 0.000 | 0.001 |
| Slc7a1 | 12 | 8032809..8108972 | 0.04 | -1.57 | -2.97 | 0.030 | 0.900 | 1.000 |
| Slc9a7 | X | 2435305..2614770 | 0.50 | -1.02 | -2.03 | 0.040 | 0.940 | 1.000 |
| Smim5 | 10 | 104451222..104457001 | 0.06 | -3.05 | -8.26 | 0.040 | 0.990 | 1.000 |
| Snph | 3 | complement(147102394..147143576) | 0.06 | -1.52 | -2.88 | 0.040 | 1.000 | 1.000 |
| Snrnp25 | 10 | complement(15686008..15689325) | 53.75 | 1.17 | 2.25 | 0.000 | 0.004 | 0.280 |
| Sntb1 | 7 | complement(95397113..95669809) | 0.58 | 1.32 | 2.50 | 0.006 | 0.490 | 1.000 |
| Soga1 | 3 | complement(153125620..153188915) | 0.13 | -1.40 | -2.65 | 0.030 | 0.860 | 1.000 |
| Sord | 3 | 114176309..114207366 | 349.50 | 1.01 | 2.02 | 0.030 | 0.850 | 1.000 |
| Sp5 | 3 | 56766475..56769277 | 0.21 | -1.49 | -2.81 | 0.030 | 0.860 | 1.000 |
| Spag4 | 3 | 151609602..151613942 | 0.03 | -4.82 | -28.31 | 0.040 | 0.970 | 1.000 |
| Spink1 | 18 | complement(38221681..38242119) | 2.93 | 1.34 | 2.53 | 0.010 | 0.610 | 1.000 |
| Spock2 | 20 | 29655226..29677926 | 0.31 | -1.77 | -3.40 | 0.010 | 0.700 | 1.000 |
| Spon2_2 | 14 | 82867946..82869792 | 0.05 | -6.13 | -69.94 | 0.010 | 0.700 | 1.000 |
| Spp1 | 14 | complement(6673686..6679901) | 0.73 | 1.39 | 2.62 | 0.009 | 0.590 | 1.000 |
| Spsb4 | 8 | complement(104840940..104912959) | 0.95 | 1.46 | 2.76 | 0.003 | 0.340 | 1.000 |
| St6galnac2 | 10 | complement(105650862..105668593) | 0.21 | -1.32 | -2.50 | 0.050 | 1.000 | 1.000 |
| Stab2 | 7 | complement(27369853..27552078) | 1.15 | -1.02 | -2.03 | 0.005 | 0.440 | 1.000 |
| Stc1 | 15 | 50891127..50903162 | 0.34 | 1.35 | 2.55 | 0.005 | 0.440 | 1.000 |
| ste2 | 14 | 22072024..22089248 | 1727.36 | 1.42 | 2.67 | 0.006 | 0.480 | 1.000 |
| Stmn3 | 3 | complement(176808017..176816162) | 0.42 | -1.58 | -2.99 | 0.020 | 0.750 | 1.000 |
| Stx1b | 1 | complement(199251844..199270627) | 0.42 | -1.10 | -2.15 | 0.050 | 1.000 | 1.000 |
| Syne4l1 | 1 | 88772904..88776999 | 0.22 | -3.00 | -7.99 | 0.007 | 0.540 | 1.000 |
| Tanc2 | 10 | 93811350..94127697 | 0.24 | -1.20 | -2.30 | 0.009 | 0.580 | 1.000 |
| Tbx20 | 8 | complement(25850962..25904570) | 0.15 | -1.56 | -2.96 | 0.030 | 0.910 | 1.000 |
| Tbx3 | 12 | complement(42480560..42492526) | 1.78 | -1.90 | -3.74 | 0.000 | 0.004 | 0.250 |
| Tcp11l1 | 3 | complement(94624071..94657377) | 0.52 | -1.22 | -2.32 | 0.010 | 0.640 | 1.000 |
| Tet3 | 4 | complement(115066309..115157263) | 0.97 | -1.38 | -2.60 | 0.001 | 0.120 | 1.000 |
| Thra | 10 | 86657285..86684933 | 2.83 | -1.42 | -2.67 | 0.000 | 0.000 | 0.005 |
| Timm21 | 18 | complement(81803173..81807627) | 5.13 | 1.19 | 2.27 | 0.001 | 0.140 | 1.000 |
| Tmem136 | 8 | complement(47396741..47404010) | 0.06 | -1.54 | -2.91 | 0.040 | 1.000 | 1.000 |
| Tmem14a_1 | 9 | 27333956..27351295 | 1.64 | -1.52 | -2.86 | 0.030 | 0.910 | 1.000 |
| Tmem170b | 17 | complement(22832641..22863966) | 0.85 | -1.00 | -2.01 | 0.040 | 1.000 | 1.000 |
| Tmem179 | 6 | complement(137073685..137084739) | 0.07 | -2.41 | -5.31 | 0.020 | 0.770 | 1.000 |
| Tmem241 | 18 | complement(3445662..3555711) | 0.38 | 1.91 | 3.76 | 0.020 | 0.750 | 1.000 |
| Tmem86a | 1 | 103172987..103177417 | 2.29 | -1.05 | -2.07 | 0.001 | 0.140 | 1.000 |
| Tmtc1 | 4 | complement(182625386..182844291) | 0.15 | -1.26 | -2.39 | 0.040 | 1.000 | 1.000 |
| Tnfaip2 | 6 | 135890931..135903647 | 5.52 | -1.14 | -2.21 | 0.003 | 0.330 | 1.000 |
| Tnfrsf19 | 15 | complement(41246308..41338284) | 0.07 | 2.91 | 7.50 | 0.010 | 0.680 | 1.000 |
| Tnfrsf9 | 5 | 168009393..168035810 | 0.58 | -2.17 | -4.51 | 0.000 | 0.010 | 0.910 |
| Tnrc18 | 12 | 13810180..13910955 | 1.69 | -1.24 | -2.37 | 0.000 | 0.040 | 1.000 |
| Tns2 | 7 | 143707237..143720595 | 4.91 | -1.05 | -2.07 | 0.002 | 0.220 | 1.000 |
| Topaz1 | 8 | 131789575..131843713 | 0.04 | 2.10 | 4.30 | 0.040 | 0.990 | 1.000 |
| Tpsb2 | 10 | 14722756..14724600 | 0.22 | 2.09 | 4.26 | 0.030 | 0.910 | 1.000 |
| Trim35 | 15 | 42960307..42976792 | 1.80 | -1.22 | -2.33 | 0.000 | 0.070 | 1.000 |
| Trim7 | 10 | 34185898..34199279 | 0.05 | -4.61 | -24.50 | 0.050 | 1.000 | 1.000 |
| Trim80 | 10 | 103992309..104004059 | 0.32 | -1.22 | -2.33 | 0.030 | 0.890 | 1.000 |
| Troap | 7 | 140758615..140766423 | 0.11 | -2.62 | -6.14 | 0.004 | 0.420 | 1.000 |
| Trpm6 | 1 | complement(234479289..234596971) | 0.70 | -1.15 | -2.23 | 0.001 | 0.110 | 1.000 |
| Tsen34l1 | 1 | complement(64024240..64030175) | 0.09 | -7.25 | -152.72 | 0.003 | 0.350 | 1.000 |
| Tspo2 | 9 | 14513037..14514416 | 0.99 | -2.35 | -5.09 | 0.001 | 0.150 | 1.000 |
| Tstd1 | 13 | 89805962..89807243 | 30.35 | 1.35 | 2.54 | 0.000 | 0.005 | 0.330 |
| Tsx | X | 74273182..74283819 | 0.98 | 1.69 | 3.23 | 0.003 | 0.340 | 1.000 |
| Ttc21a | 8 | 128622428..128657955 | 0.15 | 1.59 | 3.01 | 0.004 | 0.370 | 1.000 |
| Ttc24 | 2 | complement(187430767..187435323) | 1.46 | -1.14 | -2.21 | 0.006 | 0.490 | 1.000 |
| Tymp | 7 | complement(130342483..130347587) | 69.21 | -1.86 | -3.62 | 0.000 | 0.000 | 0.002 |
| Ubn2 | 4 | 66165845..66233230 | 0.93 | -1.12 | -2.17 | 0.004 | 0.370 | 1.000 |
| Unc80 | 9 | 73493027..73683000 | 0.02 | -1.78 | -3.44 | 0.030 | 0.870 | 1.000 |
| Upk1b | 11 | 64522130..64553228 | 0.14 | -1.99 | -3.98 | 0.020 | 0.790 | 1.000 |
| Upk3b | 12 | complement(23676152..23682381) | 0.39 | -2.10 | -4.30 | 0.010 | 0.690 | 1.000 |
| Upp2 | 3 | 44806106..44846464 | 9.48 | -1.86 | -3.63 | 0.000 | 0.020 | 1.000 |
| Ush2a | 13 | 106751625..107427329 | 0.09 | -1.27 | -2.41 | 0.003 | 0.310 | 1.000 |
| Usp49 | 9 | complement(15317413..15375365) | 0.33 | -1.34 | -2.53 | 0.005 | 0.440 | 1.000 |
| Usp9y | Y | complement(1258794..1403422) | 0.05 | 6.88 | 117.61 | 0.020 | 0.770 | 1.000 |
| Utrn | 1 | complement(6451810..6970040) | 1.84 | -1.04 | -2.06 | 0.001 | 0.110 | 1.000 |
| Vdr | 7 | complement(139342063..139394166) | 0.09 | 2.02 | 4.07 | 0.020 | 0.780 | 1.000 |
| Vps37b | 12 | 37984790..38023368 | 6.73 | 1.19 | 2.28 | 0.000 | 0.040 | 1.000 |
| Wasf1 | 20 | complement(48455705..48503898) | 0.87 | -1.02 | -2.03 | 0.010 | 0.690 | 1.000 |
| Wbp1 | 4 | complement(114817576..114820127) | 1.54 | 1.18 | 2.27 | 0.010 | 0.690 | 1.000 |
| Wbp11 | 4 | complement(170772163..170810080) | 0.66 | -2.71 | -6.54 | 0.004 | 0.380 | 1.000 |
| Wbscr27 | 12 | complement(24767351..24775891) | 3.10 | 1.01 | 2.01 | 0.020 | 0.740 | 1.000 |
| Wdr27 | 1 | complement(56808450..56942556) | 0.34 | 1.09 | 2.13 | 0.030 | 0.920 | 1.000 |
| Wdr97 | 7 | 117420788..117429504 | 0.10 | -1.21 | -2.32 | 0.050 | 1.000 | 1.000 |
| Wee1 | 1 | 174767960..174786361 | 1.93 | 1.15 | 2.22 | 0.010 | 0.710 | 1.000 |
| Wnt4 | 5 | 155649217..155672579 | 0.15 | -1.15 | -2.22 | 0.020 | 0.780 | 1.000 |
| Wt1 | 3 | 95133713..95180564 | 0.09 | -1.69 | -3.22 | 0.030 | 0.910 | 1.000 |
| Zbtb16 | 8 | complement(52985313..53146953) | 3.47 | -1.58 | -2.99 | 0.006 | 0.490 | 1.000 |
| Zbtb20 | 11 | complement(62014925..62451184) | 1.06 | -1.73 | -3.31 | 0.001 | 0.140 | 1.000 |
| Zbtb8a | 5 | complement(147554918..147584038) | 0.26 | -1.25 | -2.37 | 0.020 | 0.850 | 1.000 |
| Zdhhc23 | 11 | 61661310..61676684 | 2.29 | -1.37 | -2.59 | 0.000 | 0.002 | 0.090 |
| Zfhx3 | 19 | complement(42758127..42920344) | 0.50 | -1.45 | -2.73 | 0.002 | 0.280 | 1.000 |
| Zfp541 | 1 | 77967755..77992027 | 0.05 | -3.05 | -8.29 | 0.003 | 0.370 | 1.000 |
| Zfp704 | 2 | 94377072..94551529 | 0.34 | -1.42 | -2.67 | 0.010 | 0.660 | 1.000 |
| Zfp853 | 12 | 13323547..13328510 | 0.07 | -2.12 | -4.35 | 0.030 | 0.850 | 1.000 |
| Zfp939 | 1 | 95397991..95405285 | 0.18 | 6.01 | 64.60 | 0.040 | 0.970 | 1.000 |
| Znf750 | 10 | complement(110698891..110701137) | 0.80 | -1.38 | -2.61 | 0.002 | 0.250 | 1.000 |

Chrom, chromosome; Max group mean, Maximum group mean RPKM value
